# Strongly coloured thiocyanate frameworks with perovskite-analogue structures†

**DOI:** 10.1039/c8sc04082f

**Published:** 2018-10-26

**Authors:** Matthew J. Cliffe, Evan N. Keyzer, Matthew T. Dunstan, Shahab Ahmad, Michael F. L. De Volder, Felix Deschler, Andrew J. Morris, Clare P. Grey

**Affiliations:** Department of Chemistry, University of Cambridge Lensfield Road Cambridge CB2 1EW UK mjc222@cam.ac.uk cpg27@cam.ac.uk; Institute for Manufacturing, Department of Engineering, University of Cambridge 17 Charles Babbage Road Cambridge CB3 0FS UK; Department of Physics, University of Cambridge JJ Thomson Ave Cambridge CB3 0HE UK; School of Metallurgy and Materials, University of Birmingham Edgbaston Birmingham B15 2TT UK

## Abstract

We report the first examples of thiocyanate-based analogues of the cyanide Prussian blue compounds, M^III^[Bi(SCN)_6_], M = Fe, Cr, Sc. These compounds adopt the primitive cubic **pcu** topology and show strict cation order. Optical absorption measurements show these compounds have band gaps within the visible and near IR region, suggesting that they may be useful for applications where light harvesting is key, such as photocatalysis. We also show that Cr[Bi(SCN)_6_] can reversibly uptake water into its framework structure pointing towards the possibility of using these frameworks for host/guest chemistry.

## Abstract 2

We report the first examples of thiocyanate-based analogues of the cyanide Prussian blue compounds, M^III^[Bi(SCN)_6_], M = Fe, Cr, Sc.

## Graphical abstract

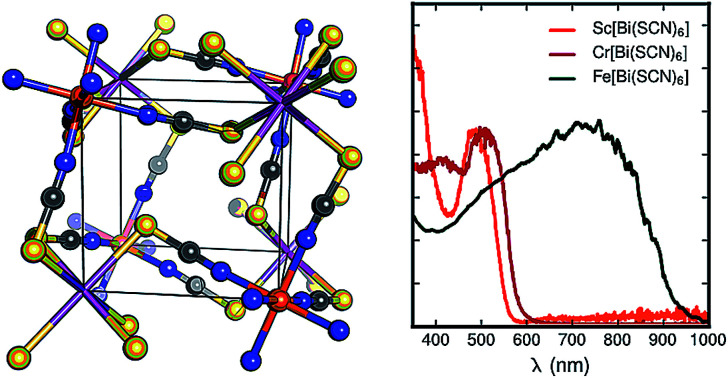

Molecular framework materials, where metal centres are connected by molecular ligands into three dimensional networks, bridge the gap between the inorganic and organic solid state. Perhaps the most widely studied inorganic framework structure-type is the ABX_3_ perovskite, which is formed from a three dimensional, topologically cubic, network of corner sharing BX_3_ octahedra, charge balanced by A-site guests occupying the network cavities. Perovskites are important because of the wide range of physical properties that they can support. The relatively open structure permits ferroelectricity *via* the correlated displacement of cations and the facile (de)intercalation of guests (as in WO_3_ electrochromic devices^1^), and the 3D cubic BX_3_ connectivity facilitates correlated behaviour, including multiferroicity,^2^ octahedral-tilt driven ferroelasticity^3^ and the extended electronic states found in heavy metal halide solar cell absorbers.^4^ Molecular framework perovskites are of particular interest as the large ‘X’ ligands permit both unusual flexibility impossible in inorganic perovskites (*e.g.* tilt-driven ferroelectricity^5,6^) and a more extensive range of guest chemistry.^7^ The properties of these materials are already of great interest, notably the Prussian blue metal cyanides, which are useful as both catalysts^8^ and electrochemical components,^9^ and the alkylammonium metal formates, which can possess simultaneous dipolar and magnetic order.^10^ Despite the promise of molecular perovskites, the range of chemistry is limited relative to that of their ‘atomic’ counterparts: most work is confined to studies of the first-row transition metals and their complexes with a small number of ligands.^7^

Exploring the chemistries of underexploited metal cations and molecular ligands thus offers the opportunity to produce new materials with properties very different from the current exemplars. Thiocyanate (SCN^−^) is a particularly interesting candidate small ligand for the assembly of molecular frameworks, as its chemical softness allows easy access to a wider range of coordination chemistry. In addition, thiocyanate based materials often have strong optical absorption, for example the terpyridine Ru(SCN)_3_ complexes used in dye-sensitised solar cells,^11^ and can contain reasonably strong magnetic interactions.^12,13^ Despite the potential of this ligand, there are relatively few reported structures of three dimensional thiocyanate based frameworks, of which only two are perovskites, CsCd(SCN)_3_ and the double perovskite (NH_4_)_2_ CdNi(SCN)_6_.^14–18^ In addition, there are no known thiocyanate analogues of the Prussian blue structured (*i.e.* A-site vacancy double perovskite) cyanides.

One reason why there are fewer thiocyanate-based framework materials than for other small molecular ligands such as formate, azide or cyanide, are its stringent bonding requirements. Unlike other widely investigated small ligands, the coordinative preferences of the two termini of the SCN^−^ ligand are quite distinct: the S-terminus is soft and binds to Class B metals and the N-terminus is harder and binds to Class A metals. As forming a framework requires both ends of the ligand to coordinate to a metal, this means a simple homometallic framework will have to have intermediate hardness. There are very few metals that have been shown to form homoleptic complexes with both the S and N-termini of the SCN^−^ ligand and of these, only Cd^2+^ has been investigated for its framework forming ability [Fig. 1(c)].^17,18^ Not only does this limit the range of possible compounds, as a d^10^ cation, Cd^2+^ is inherently unpromising for optical and magnetic applications. Double perovskite derived structures, where two different metal cations fulfil the differing bonding requirements of the two termini, offer one route for expanding the compositional space of thiocyanate.

**Fig. 1:**
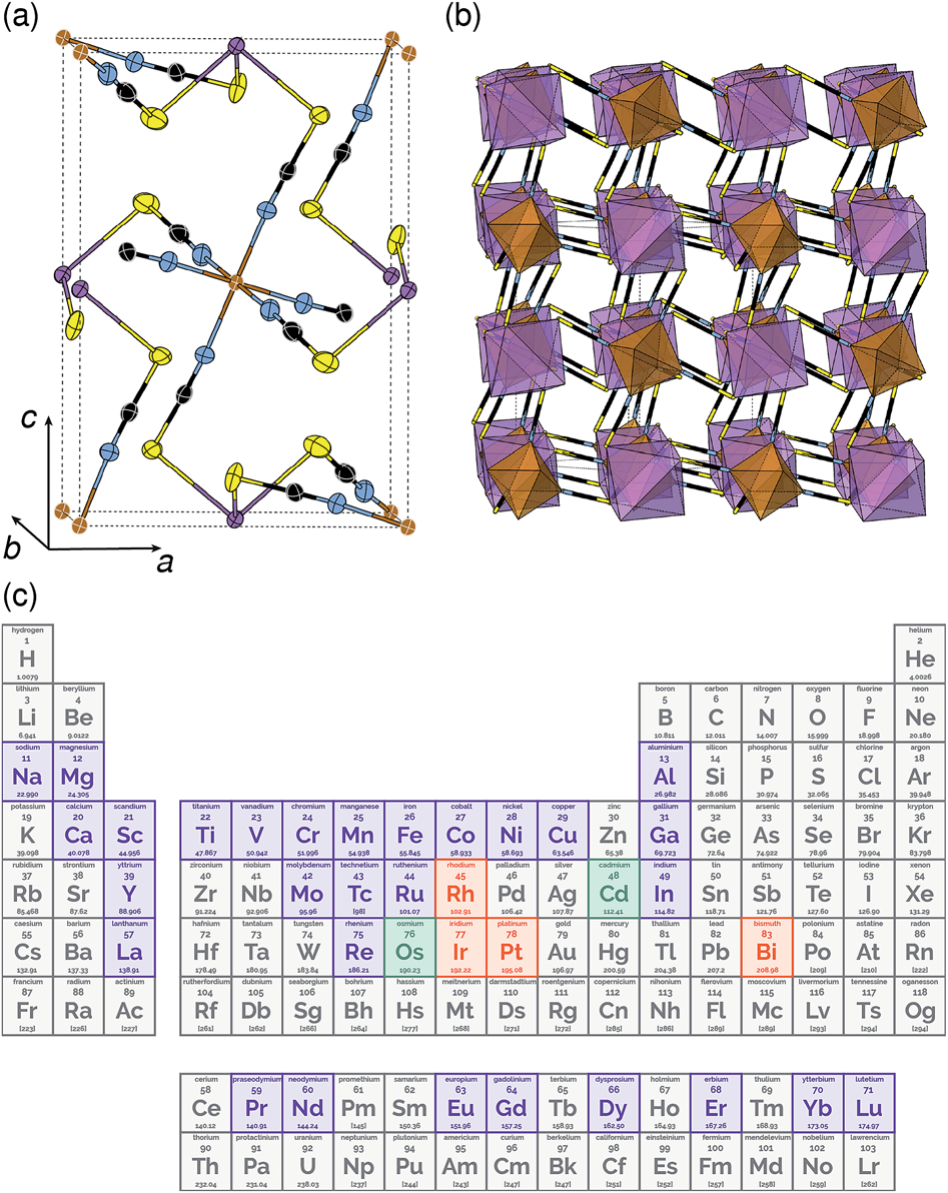
Crystal structure of Fe[Bi(SCN)_6_] in (a) ORTEP and (b) polyhedral representations. Atoms are coloured as follows Bi, purple; Fe, brown; S, yellow; C, black and N, blue. (c) The periodic table coloured by whether the homoleptic metal hexathiocyanate anion is known. If [M(NCS)]^*n*−^ is known in the CSD or ICSD structural database, the element is coloured indigo, if [M(NCS)]^*n*−^ is known, it is coloured orange, if both are known, it is coloured green.

Out of the metals which preferentially coordinate to S-terminus of NCS^−^ Bi^3+^ is perhaps the most attractive option, as it is non-toxic and inexpensive. Previous work has established that the homoleptic octahedral [Bi(SCN)_6_]^3−^ anion can be used to form framework structures, in the alkali metal double hexagonal perovskite Cs_2_Na[Bi(SCN)_6_] and the nickel arsenide structured Ln[Bi(SCN)_6_]·*x*H_2_O, *x* = 3–5, Ln = La–Dy.^14,16^ In addition, [Bi(SCN)_3_]^3−^ has been reported to form an insoluble compound suitable for a quantitative chemical analysis with chromium(iii) solutions.^19^ A further interesting feature of these bismuth thiocyanate frameworks are their yellow and orange colours, also found in bismuth thiocyanate hemihydrate 
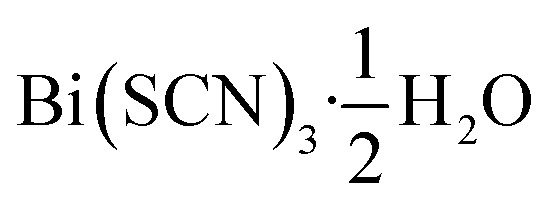
.^20^ There are, however, no reported structures or physical properties of either cubic perovskite derived structures or transition metal based bismuth thiocyanate frameworks.

In this paper we report the synthesis, structure and optical properties of three analogues of the cyanide Prussian blues, the thiocyanate frameworks M^III^[Bi(SCN)_6_]; M = Fe, Cr and Sc, each of which is strongly coloured. We find that these materials form isotypic structures with large octahedral tilts and demonstrate that Cr[Bi(SCN)_6_] can reversibly take up water.

## 1 Experimental section

### 1.1 Synthesis of HSCN

In a 250 mL round bottom flask, NH_4_SCN (5 g, 65.7 mmol) was dissolved in 5 mL H_2_O and cooled to 0 °C in an ice bath. A H_2_SO_4_ solution (*ca.* 7 mL of H_2_SO_4_ in 12 mL H_2_O) was then added dropwise to the cooled NH_4_SCN solution. The reaction mixture was stirred for 30 min before being warmed to room temperature. The aqueous mixture was subsequently extracted with diethyl ether (2 × 20 mL) and the organic phase was retrieved and its volume reduced by half using a stream of N_2_.

### 1.2 Synthesis of H_3_[Bi(SCN)_6_] solution and Bi(SCN)_3_

The synthesis of H_3_[Bi(SCN)_6_] was adapted from ref. 20. Bi_2_O_2_(CO_3_) (0.50 g, 0.98 mmol) was suspended in *ca.* 12 mL H_2_O followed by the addition of the HSCN/ether solution. The resulting reaction mixture was stirred vigorously under a slight flow of N_2_ until all ether had been removed and the solution had turned bright orange. Any remaining solids were filtered off and the orange solution was placed under a slight vacuum to remove any excess HSCN. A portion of the H_3_[Bi(SCN)_6_] solution was left to evaporate yielding large, yellow, crystals of Bi(SCN)_6_.

### 1.3 Synthesis of Fe[Bi(SCN)_6_]

Fe[Bi(SCN)_6_] can synthesised *via* a number of different routes leading to a variety of morphologies.

#### 1.3.1 Single crystals of Fe[Bi(SCN)_6_]

Approximately 10 mL of the H_3_[Bi(SCN)_6_] solution synthesised as described above was added to Fe_2_O_3_ (1 mmol, 159.7 mg), and then stirred for 1 h. Excess Fe_2_O_3_ was then filtered off under vacuum, and the deep red solution was left to slowly evaporate over a few days, yielding diffraction quality very dark green single crystals of Fe[Bi(SCN)_6_].

#### 1.3.2 Microcrystalline Fe[Bi(SCN)_6_]

KSCN (15 mmol, 1.458 g) was added to a stirred suspension of Bi(NO_3_)_3_·5H_2_O (2.5 mmol 1.213 g) in 100 mL of butanone, producing a yellow-orange solution and a white precipitate of KNO_3_. Fe^III^(NO_3_)_3_·9H_2_O (2.5 mmol, 1.010 g), was then added, producing a deep purple solution. The reaction mixture was stirred for overnight and then filtered to remove KNO_3_, yielding a very deep purple solution of Fe[Bi(SCN)_6_]. This solution was evaporated to dryness, and then extracted using 100 mL of dry tetrahydrofuran. After removal of the solvent *in vacuo* and drying at 50 °C for 2 h, a very dark green microcrystalline powder of Fe[Bi(SCN)_6_] was produced (1.02 g, 67%).

### 1.4 Synthesis of Sc[Bi(SCN)_6_]

The route used to synthesise microcrystalline Fe[Bi(SCN)_6_] was adapted for the synthesis of Sc[Bi(SCN)_6_]. KSCN (0.6 mmol, 0.0583 g) was added to a stirred suspension of Bi(NO_3_)_3_·5H_2_O (0.1 mmol 0.0485 g) in 10 mL of butanone producing a yellow-orange solution and a white precipitate of KNO_3_. Sc^III^(NO_3_)_3_·*x*H_2_O (0.1 mmol, 0.023 g), was then added, producing a deep orange solution. The reaction mixture was stirred for overnight and then filtered to remove KNO_3_, yielding an orange solution of Sc[Bi(SCN)_6_]. This solution was evaporated to dryness from which diffraction quality single crystals of Sc[Bi(SCN)_6_] could be obtained.

### 1.5 Synthesis of Cr[Bi(SCN)_6_]

(NH_4_)_3_[Cr(NCS)_6_] (1 mmol, 455 mg), prepared according to literature methods,^21^ was dissolved in 2 mL distilled water and slowly added to a solution of Bi(NO_3_)_3_·5H_2_O (1 mmol, 485.1 mg) in 2 mL 3 N aqueous HNO_3_, yielding a brick red precipitate. The product was then filtered under vacuum and washed with 10 mL H_2_O, and dried at 50 °C, yield (74.0%). The product was analysed by elemental analysis (combustion analysis for CHN and ICP for Cr and S). Calculated (found) CrBiS_6_C_6_N_6_: Cr 8.5% (7.79%), S 31.5% (29.87%), C 11.8% (11.44%), N 13.8 (13.44%), H 0.0% (0.0%). Cr[Bi(SCN)_6_]·H_2_O is formed when this synthesis is carried out using 3 N HNO_3_ as a solvent for (NH_4_)_3_[Cr(NCS)_6_] and when Cr[Bi(SCN)_6_] is exposed to ambient humidity over a period of weeks.

### 1.6 Thermogravimetric analysis

Thermogravimetric analysis was carried out with a Mettler Toledo TGA/SDTA 851 thermobalance. Powder samples of 20–40 mg were placed in a 100 μL Al_2_O_3_ crucible and heated to 650 °C (at 10 °C min^−1^) under a flow of N_2_.

### 1.7 Optical measurements

Diffuse reflectance measurements were carried out on finely ground powdered samples, diluted to 10 wt% with BaSO_4_ to remove effects of strong absorption, using a Varian Cary 50 UV-vis spectrometer equipped with a diffuse reflectance accessory (DRA) probe (Barrelino, Harrick Scientific) measured over the range 350–1000 nm. The optical absorption measurements were performed using an integration sphere equipped PerkinElmer lambda 750 UV-vis-NIR commercial setup. The set-up includes light source of tungsten-halogen (for vis-NIR region) and deuterium (for UV region) lamps, a conventional photomultiplier (PMT) detector, a 10 cm integrating sphere module attachment, a monochromator, and a detector slit width of 10 nm. All measurements were performed under standard ambient conditions. Films were prepared by drop-casting the crystals, dissolved/suspended in the appropriate solvent (DMF), on the quartz/glass slide substrate. The transmitted light was corrected using a reference of an empty quartz coverslip of the same type and thickness as the substrate used for the sample. The absorption onsets were determined by extrapolation from a Tauc plot. We used our DFT results to guide our assumed Tauc exponents: for Cr[Bi(SCN)_6_] and Sc[Bi(SCN)_6_] we carried out fits for both direct and indirect band gaps and for Fe[Bi(SCN)_6_] we used the Tauc exponent for a direct band gap. Further details of the optical characterisation can be found in the ESI.†

### 1.8 Powder X-ray diffraction

All microcrystalline samples were initially assessed for their purity *via* their X-ray powder diffraction patterns, measured using a PANalytical Empyrean diffractometer (Cu Kα radiation, *λ* = 1.541 Å). High resolution synchrotron measurements on Cr[Bi(SCN)_6_] were carried out at beamline 11-BM at the Advanced Photon Source (APS) using a wavelength of 0.414537 Å and on Cr[Bi(SCN)_6_]·H_2_O at beamline I11 at the Diamond Light Source, using a wavelength of 0.826168 Å.^22,23^ Variable temperature powder diffraction measurements on Cr[Bi(SCN)_6_]·H_2_O were carried out at beamline I11 at the Diamond Light source, using a hot air blower, and on Cr[Bi(SCN)_6_] on a PANalytical Empyrean diffractometer using a Anton Parr XRK 900 furnace. Analysis of all powder diffraction data (including indexing, Pawley refinement and Rietveld refinement) was carried out using the TOPAS Academic 4.1 structure refinement software package.^24–26^ Full details of the powder X-ray refinements can be found in the ESI.†

### 1.9 Single crystal X-ray diffraction

Single-crystal X-ray diffraction data were collected using a Nonius KappaCCD diffractometer, using graphite monochromated Mo Kα radiation (*λ* = 0.7107 Å). Structure solution was carried out using SHELXT and refinement with SHELXL, within the OLEX2 graphical interface.^27–29^ All non-hydrogen atoms were refined anisotropically with no additional restraints or constraints. Supplementary crystallographic data for this paper including of all single crystal structures can be found in the ESI† and were deposited in the CCDC 1860296–1860300.

### 1.10 DFT calculations

Geometry optimised structures and associated spectral properties were calculated using density-functional theory (DFT), using the structure of Fe[Bi(SCN)_6_] determined from single crystal diffraction as a starting model. Calculations were performed using the plane wave CASTEP DFT code,^30^ and the Perdew–Burke–Ernzerhof (PBE) exchange–correlation functional was used with Vanderbilt ultrasoft pseudopotentials.^31^ A basis set containing plane waves with energies of up to 800 eV and a Monkhorst–Pack (MP) grid corresponding to a Brillouin zone (BZ) sampling grid finer than 2π × 0.03 Å^−1^ was used. Geometry optimisations were performed with spin polarisation and spectral calculations were additionally performed with DFT+*U*. To determine the influence of the value of *U* on the electronic structure, separate calculations were performed with *U* = 0 and 2.5–5.5 eV for the Cr d orbitals and *U* = 0 and 4.0–6.0 eV for the Fe d orbitals in 0.5 eV steps. Changing *U* values had little impact on band occupancy and the overall structure of the bands, however by changing the energy of the metal d orbitals it was possible to find values of *U* that reproduced the experimentally observed band gaps, *U* = 2.5 eV and *U* = 4.5 eV were chosen for the Cr and Fe d-orbitals respectively. These values are broadly consistent with *U* values found in previous studies.^32,33^ Density of states for both materials was calculated using OptaDOS^34,35^ using adaptive broadening.^36^ Additional information on the calculations, and particularly the variation in *U* can be found in the ESI.†

## 2 Results and discussion

All three compounds were synthesised by reacting thiocyanatobismuthate solutions, in dilute nitric acid or polar aprotic solvents, with a transition metal source. The key difference between the synthetic routes required for these materials was due to the large difference in solubilities. Cr^III^[Bi(SCN)_6_] is soluble only in coordinating aprotic solvents such as DMF or DMSO, whereas Sc^III^[Bi(SCN)_6_] and Fe^III^[Bi(SCN)_6_] are soluble in much more weakly coordinating solvents including diethyl ether. Cr^III^[Bi(SCN)_6_] was therefore prepared by precipitation from acidic aqueous solution whereas Fe^III^[Bi(SCN)_6_] and Sc^III^[Bi(SCN)_6_] were prepared by evaporation from weakly coordinating solvents. Due to the slower rate of crystallisation of Fe^III^[Bi(SCN)_6_] and Sc^III^[Bi(SCN)_6_] it was possible to grow diffraction quality single crystals, from which we were able to solve their structures. These structures were used as the starting points for Rietveld refinement of synchrotron powder diffraction data of Cr^III^[Bi(SCN)_6_], confirming that despite the differences in solubility, which we ascribe to the kinetic inertness of Cr^III^ complexes, all three compounds adopt isotypical structures consisting of M^III^N_6_ and Bi^III^S_6_ octahedra linked by thiocyanate ligands into a 3D network with **pcu** topology [Fig. 1a and b]. This structure is both analogous to the cyanide Prussian blue structure and can also be described as a double perovskite structure with vacancies occupying the A-site.

**Fig. 2:**
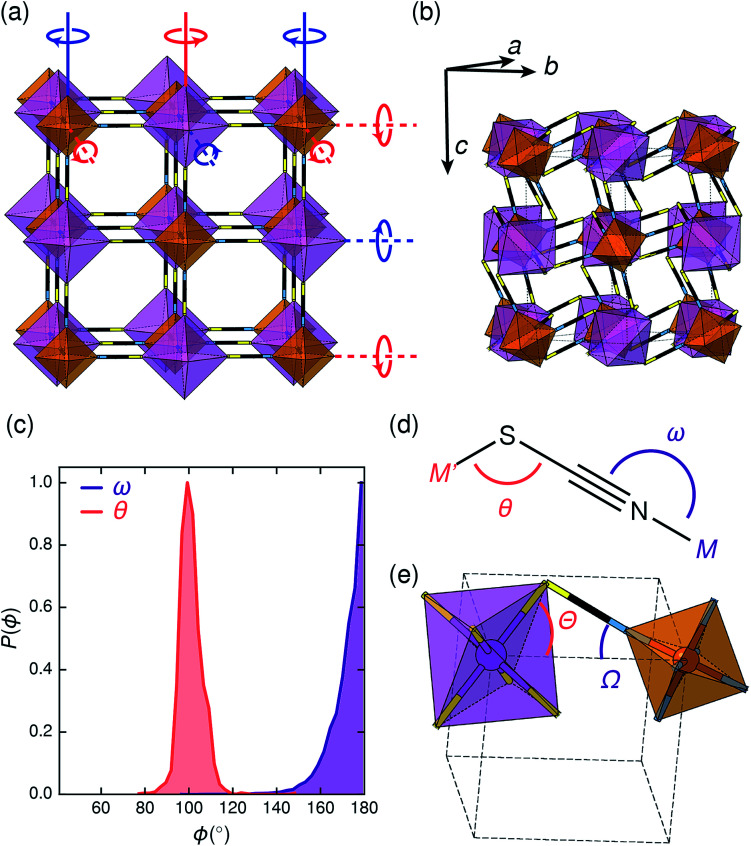
(a) Illustration of the tilt system of Fe[Bi(SCN)_6_] shown on the hypothetical *Fm*3̄*m* aristotype: red indicates clockwise and blue indicates anticlockwise. In-phase (*k* = 0) tilts are indicated by solid lines, anti-phase tilts (*i.e.*
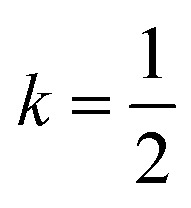
) are indicated by dashed lines. (b) The actual crystal structure of Fe[Bi(SCN)_6_] shown at the same scale. Its unit cell is shown by black, dashed lines. (c) Distribution of the M–X–C angle for N- and S-bound thiocyanate, determined *via* database search in the CSD. There is a clear preference for linear M-NCS and bent M-SCN bonds. (d) Definition of relevant bond and tilt angles. Pseudo-cubic unit cell shown in dashed lines.

Unlike the cyanide-based Prussian blue materials, which crystallise with high symmetry cubic structures and often have substantial cation and anion disorder,^37^ these thiocyanate frameworks are well-ordered and adopt distorted monoclinic structures. The size of the distortion is demonstrated by greatly reduced volume compared to the hypothetical cubic *Fm*3̄*m* aristotype, which is 54% for Fe[Bi(SCN)_6_] [Fig. 2a and b]. This distortion means that the structures are much less porous than might be expected from consideration of the length of the NCS^−^ ligand alone.

Symmetry analysis carried out using the ISODISTORT software^38^ showed that the two most important symmetry-adapted distortion modes which relate this monoclinic structure to the face-centered cubic parent structure are the Γ_4_^+^ and X_3_^+^ modes. These two modes are, to a good approximation, tilts of rigid metal octahedra. The X_3_^+^ mode corresponds to an in-phase tilt along *c*, *i.e.* the tilts in every plane perpendicular to *c* are in the same sense. The Γ_4_^+^ mode corresponds two equal sized out-of-phase tilts along the *a* and *b* directions, *i.e.* the tilts in sequential planes perpendicular to *a* and *b* directions are in opposite senses. The octahedral tilts pattern can therefore be summarised by the *a*^−^*a*^−^*c*^+^ Glazer notation (conventionally *a*^+^*b*^−^*b*^*−*^) [Fig. 2a].^39^ This means that perhaps surprisingly, these very distorted structures adopt the most common tilt system for double perovskites,^40^ and that non-standard cooperative perovskite distortions such as anti-phase tilts and columnar shifts, although common in other molecular perovskite-type frameworks, do not play a significant role in these structures.^5,41^

The origin of tilting in both inorganic and molecular perovskite structures is ordinarily the size mismatch between the A-site cation and the pore size of the anionic frameworks.^40,42^ Prussian blue structured materials, like these M^III^[Bi(SCN)_6_] frameworks, have no A-site cation and a neutral framework and so ‘tolerance factor’ arguments cannot be used. Instead, the size of the tilts is largely driven by the general tendency towards higher density structures and the geometry of ligand bonding: in metal thiocyanate frameworks the M–N–C bonds are approximately linear (*ω* ≈ 180°) and the M′–S–C bonds are bent (*θ* ≈ 100°) [Fig. 2(c)]. The linear M–N–C angle, stabilised by the ligand field of the transition metal, means that the M(NCS)_6_ unit behaves as a larger rigid octahedron, preventing the structure from undergoing unconventional tilting. Symmetry analysis of the only other reported examples of perovskite-derived thiocyanate-frameworks demonstrates the importance of linear M–N–C bonding: (NH_4_)_2_ Ni[Cd(SCN)_6_], which has Ni(NCS)_6_ octahedra, has tilts which belong to the conventional *a*^+^*b*^−^*b*^−^ tilt system whereas CsCd(SCN)_3_, which has more flexible Cd(NCS)_6_ octahedra with bent Cd–N–C bond angles (as small as 116.5°), has more complex cooperative distortions, including unconventional tilts.^5^

The shapes of the molecular orbitals of the thiocyanate ligand also explain the magnitude of the observed tilts in these frameworks. There is no unique way of decomposing the total tilts into separate rotations around each of the three axes, as the octahedral rotations do not commute.^39^ While this effect is small for small (<10°) tilts, it cannot be neglected for larger tilts. A good approximate decomposition of the octahedral rotations into separate tilt angles can be found in the angles between the axes of the metal octahedron, *i.e.* the metal–ligand bonds, and the pseudo-cubic unit cell axes, defined by the vectors which connect the metal centres [Fig. 2d and e]. For Fe[Bi(SCN)_6_] at 180 K, the FeN_6_ tilt angles are *Ω*_[001]_ = 24.0°, *Ω*[11̄0] = 27.8° and *Ω*_[110]_ = 24.4° giving an average tilt angle of 〈*Ω*〉 = 25.4°. The corresponding tilt angles for the BiS_6_ octahedra are larger: *Θ*_[001]_ = 51.6°, *Θ*[11̄0] = 53.2° and *Θ*_[110]_ = 53.3°, which give an average tilt angle of 〈*Θ*〉 = 52.7°. As the M–N–C bond angle is nearly linear, the sum of the tilts of the BiS_6_ and MN_6_ octahedra relates directly to the Bi–S–C bond angle (*Θ* + *Ω* ≈ 180° − *θ*).

The scale of the octahedral tilts in NCS perovskite-derived structures is therefore set by the bent Bi–SCN bonding, rather than the mismatch between a guest cation and the framework. The tilt-angles are therefore likely to be only weakly dependent on temperature, and so thermal second-order octahedral-tilt driven phase transitions are not expected to be common in this series of compounds.

As anticipated, therefore, variable temperature X-ray diffraction experiments confirmed that both Fe[Bi(SCN)_6_] and Cr[Bi(SCN)_6_] remain in this monoclinic structure at all temperatures measured: single crystal diffraction on Fe[Bi(SCN)_6_] shows no phase transitions between −93 and 102 °C within 30° of its decomposition temperature [ESI Fig. 20†], and variable temperature PXRD studies on Cr[Bi(SCN)_6_] show that it remains isostructural from room temperature up to 300 °C, where it decomposes. The single crystal structures of Fe[Bi(SCN)_6_] at different temperatures show that the changes in tilt angles are comparatively small compared to their overall magnitude: they decrease by less than 1° over 200 K (at 180 K 〈*Ω*〉 = 25.4° and 〈*Θ*〉 = 52.5°, at 375 K 〈*Ω*〉 = 25.2° and 〈*Θ*〉 = 52.0° [Fig. 2(e)]).

**Fig. 3:**
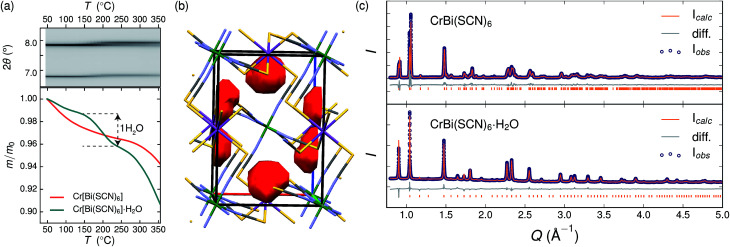
(a) Dehydration of Cr[Bi(SCN)_6_]·H_2_O between 150 °C and 200 °C. Top panel: variable temperature PXRD measurements of Cr[Bi(SCN)_6_]·H_2_O, highlighting the separation of the {110} and (002) reflections at around 2*θ* = 7.8°. Bottom panel: TGA measurements on hydrous (teal) and anhydrous (orange) show the presence of a step at around 170 °C in the hydrated sample, but no similar step in the dehydrated sample. (b) Structure of Cr[Bi(SCN)_6_]·H_2_O determined from powder diffraction data, with guests removed. The void space within the cell is shown in red (contact surface determined using Mercury, probe size 1.2 Å). Colour scheme as follows: Bi, purple; Cr, green; N, blue; C, black and S, yellow. (c) Rietveld refinement of synchrotron powder diffraction data for both hydrated and anhydrous Cr[Bi(SCN)_6_].

Variable temperature powder diffraction investigations also explained the origin of some of the unusual sample variability in Cr[Bi(SCN)_6_] [Fig. 3]. Despite its apparently dense structure Cr[Bi(SCN)_6_] can take up water into its structure forming a hydrate, Cr[Bi(SCN)_6_]·H_2_O. This hydrate is formed when the synthesis of Cr[Bi(SCN)_6_] is carried out in more acidic solution, and also slowly forms from Cr[Bi(SCN)_6_] at room temperature and ambient humidity over a period of weeks and when Cr[Bi(SCN)_6_]·H_2_O is heated above 150 °C, this guest water is lost, leading to a reduction in cell volume by 19 Å^3^ and a mass loss of 3% [Fig. 3(a)]. Further experiments will be required to assess how stable this material is to extended cycling of water uptake/release. The clearest diffraction signature of the dehydration is the separation of the {110} and (002) reflections, which are near coincident in the hydrate, but do not overlap in the anhydrate [Fig. 3(c)]. As with the other M[Bi(SCN)_6_] frameworks, this apparent peak overlap does not correspond to any change in framework symmetry, but merely results from the near cubic pseudo-symmetry of the metal substructure.

Due to the relatively small contribution of the guest water to the total electron density (3%) and peak broadening resulting from the small crystallite sizes, it was not possible to localise the guest water from the fourier difference map generated from Rietveld refinement of Cr[Bi(SCN)_6_]·H_2_O. Calculation of the geometric voids in the empty framework structure did however reveal a single void capable of containing a water molecule [Fig. 3(b)]. Rietveld refinement of the framework structure including an oxygen atom situated in that void with its occupancy fixed to the value derived from TGA measurements produced a small improvement in the quality of fit compared to the empty framework. The limitations of the powder diffraction data prevented us from deriving further information about the location of the guest water molecule. This hydrate can therefore be considered to be an intermediate member between the full perovskites NH_4_Ni[Cd(SCN)_6_] and Cs_2_Cd[Cd(SCN)_6_], which contain cationic guests, and the other empty Prussian blue structures we report here, with the A-site occupied half by vacancies and half by water: *i.e.* □(H_2_O)Cr[Bi(SCN)_6_].

**Fig. 4:**
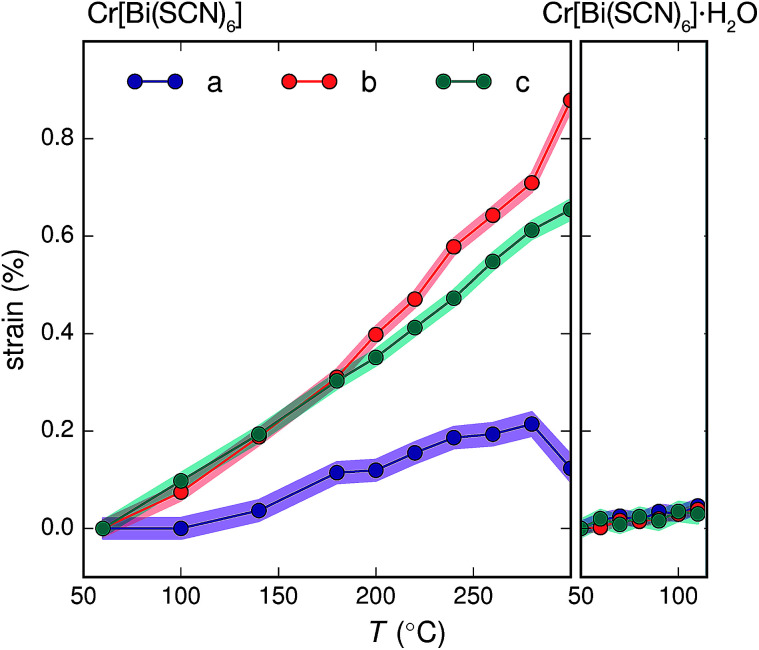
Crystallographic strain of Cr[Bi(SCN)_6_] and Cr[Bi(SCN)_6_]·H_2_O along the unit cell axes determined *via* variable temperature powder X-ray diffraction. The total change in volume for Cr[Bi(SCN)_6_] over this region (1.7%) is significantly smaller than the volume difference between the hypothetical aristotype and the room temperature structure (54%). As the changes in sin (*θ*) are two orders of magnitude smaller than the strains along the unit cell axis directions the strain eigenvalues are to a good approximation equal to the unit cell axis strains.

The presence of guest water also affects the thermomechanical properties of the Prussian blue framework [Fig. 4]. Whereas Fe[Bi(SCN)_6_] and Cr[Bi(SCN)_6_] have coefficients of thermal expansion comparable to that of the more rigid members of the formate perovskite family, hydrated Cr[Bi(SCN)_6_]·H_2_O is a factor of four stiffer again [Table 1].^43^ The rigidifying effect of guests has been noted in both inorganic and molecular framework materials, where even weakly interacting guests can reduce the material's flexibility.^44,45^ The maximum thermal expansion seen in Cr[Bi(SCN)_6_], of 1.7% over 250 °C range, is smaller than both the volume change on water uptake (2.3%) and particularly the volume change relative to the cubic structure aristotype (54%). This suggests that octahedral tilt transitions to higher symmetry structures are unlikely to occur in this family on heating, even if framework decomposition can be avoided.

**Table 1:** Coefficients of thermal expansion for M[Bi(SCN)_6_]

	*α*_1_ (MK^−1^)	*α*_2_ (MK^−1^)	*α*_3_ (MK^−1^)	*α*_*V*_ (MK^−1^)
Fe[Bi(SCN)_6_]^a^	−6(2)	41.4(3)	21.3(6)	57(2)
Cr[Bi(SCN)_6_]^b^	9.5(1.4)	35(2)	27.6(6)	69(4)
Cr[Bi(SCN)_6_]·H_2_O^c^	6.2(1.3)	6.2(1.7)	4.5(1.4)	16.9(1.7)

a Determined over range −93 to 102 °C.

b Determined over the range 60 to 280 °C.

c Determined over range 50 to 110 °C. Estimated standard errors in parentheses.

**Fig. 5:**
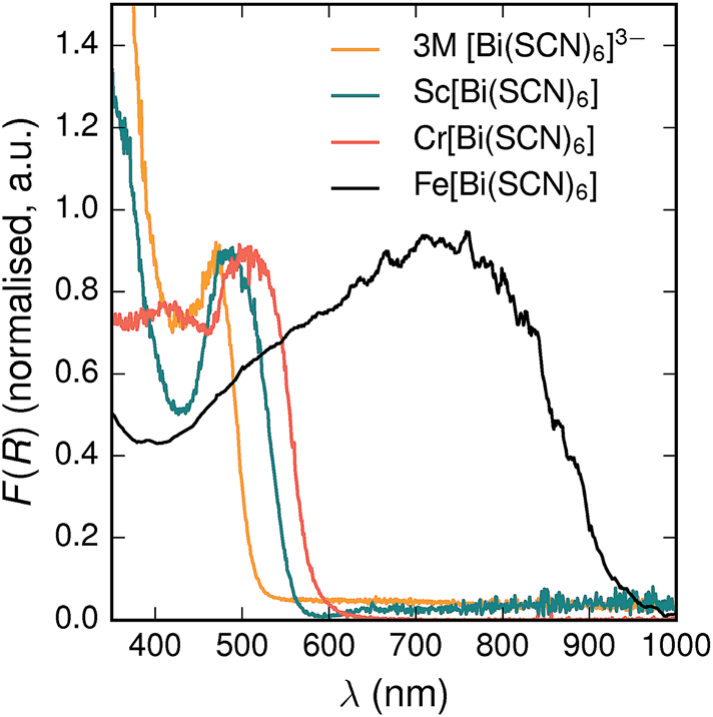
Diffuse reflectance measurements of powdered samples of M^III^[Bi(SCN)_6_] materials and a concentrated solution of [Bi(SCN)_6_]^3−^.

All three materials, Sc[Bi(SCN)_6_], Cr[Bi(SCN)_6_] and Fe[Bi(SCN)_6_] are strongly coloured (respectively, orange-red, brick red and black). Diffuse reflectance spectroscopy and absorption spectroscopy on powder samples confirmed that these materials possess band gaps determined from a Tauc plot within the visible and near infra-red region [Table 2, Fig. 5]. The variation in the observed absorption spectra shows that both bismuth-thiocyanate and transition metal-thiocyanate moieties play a role in the optical properties of the material. Transition metal thiocyanate complexes, particularly Fe(iii) thiocyanate complexes, are well known for their intense ligand-to-metal charge transfer (LMCT) bands, from primarily S 3p and N 2p states to metal 3d states, and likewise Bi^3+^ thiocyanate complexes have been reported to absorb strongly, both in the solid state^20^ and in concentrated solutions of bismuth thiocyanate (*λ*_max_ = 472(3) nm), due to LMCT to the empty Bi 6p states. The relevance of this LMCT to bismuth for these M[Bi(SCN)_6_] is shown by Sc[Bi(SCN)_6_] which will not have any metal d–d electronic transitions. The observed absorption maximum Fe[Bi(SCN)_6_] occurs at significantly lower energies (*λ*_max_ = 740(20) nm) than observed for Fe(iii) thiocyanate in solution (*λ*_max_ = 480(20) nm). This indicates that the observed optical gap cannot be explained by the considering isolated Fe(NCS)_6_^3−^ moieties, but rather must be influenced by its structure in the solid-state framework. This difference between solution and solid-state spectra has also been reported for alkyammonium salts of the hexakisisothiocyanato iron(iii) complexes, suggesting that coordination to bismuth in particular is not responsible for the observed red-shift.^46,47^

**Table 2:** Experimental and DFT-derived band gaps^a^

*E*_g_ (eV)	Expt.		DFT+*U*	
	Direct	Indirect	Direct	Indirect
Sc[Bi(SCN)_6_]	2.25(5)	2.13(5)	—	—
Cr[Bi(SCN)_6_]	2.18(5)	2.04(5)	2.15	1.89
Fe[Bi(SCN)_6_]	1.20(5)	—	1.18	1.14

a Estimated standard errors in parentheses. *U* = 2.5 eV for Cr[Bi(SCN)_6_] and *U* = 4.5 eV for Fe[Bi(SCN)_6_].

To understand better the optoelectronic properties of these materials we therefore carried out density-functional theory (DFT) calculations on Fe[Bi(SCN)_6_] and Cr[Bi(SCN)_6_]. We calculated the electronic band structures using spin-polarised GGA as implemented by CASTEP on geometry-optimised structures, and then used OptaDOS to determine from these results the optical band gap and the electronic density of states, including the atom and angular momentum projected density of states. The calculated band gaps were significantly smaller than those observed experimentally. This is commonly observed in DFT calculations due to the self-interaction error, which is particularly pronounced for the contracted d orbitals. We therefore repeated the calculations of the electronic structure, but now also including a Hubbard *U* in the Hamiltonian, which we systematically varied. We found that introducing this additional factor did not perturb qualitative features of the electronic structure aside from the energy of the transition metal d states. These calculations allowed us to determine that for both Fe[Bi(SCN)_6_] and Cr[Bi(SCN)_6_] the smallest electronic transitions are indirect, with the valence band maximum at the *Γ* point and the conduction band minimum at the *D* point, 
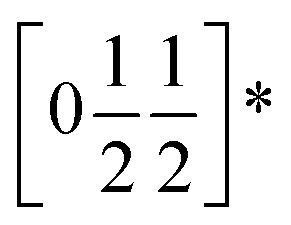
. The difference between the direct and indirect gaps in both cases was small, with Δ*E*_g_ = 0.2 eV for Cr[Bi(SCN)_6_] and even smaller for and Δ*E*_g_ = 0.04 eV for Fe[Bi(SCN)_6_]. We therefore fitted the absorption edge using Tauc plots in both the direct and indirect forms for Cr[Bi(SCN)_6_] and Sc[Bi(SCN)_6_]. The difference between direct and indirect band gaps is sufficiently small for Fe[Bi(SCN)_6_] (<2*kT*) that for this compound we measured the band gap using the direct form of the Tauc plot. The calculations were able to reproduce the experimentally observed band gaps for both Cr[Bi(SCN)_6_] and Fe[Bi(SCN)_6_] with physically reasonable values of *U* [Table 2].

**Fig. 6:**
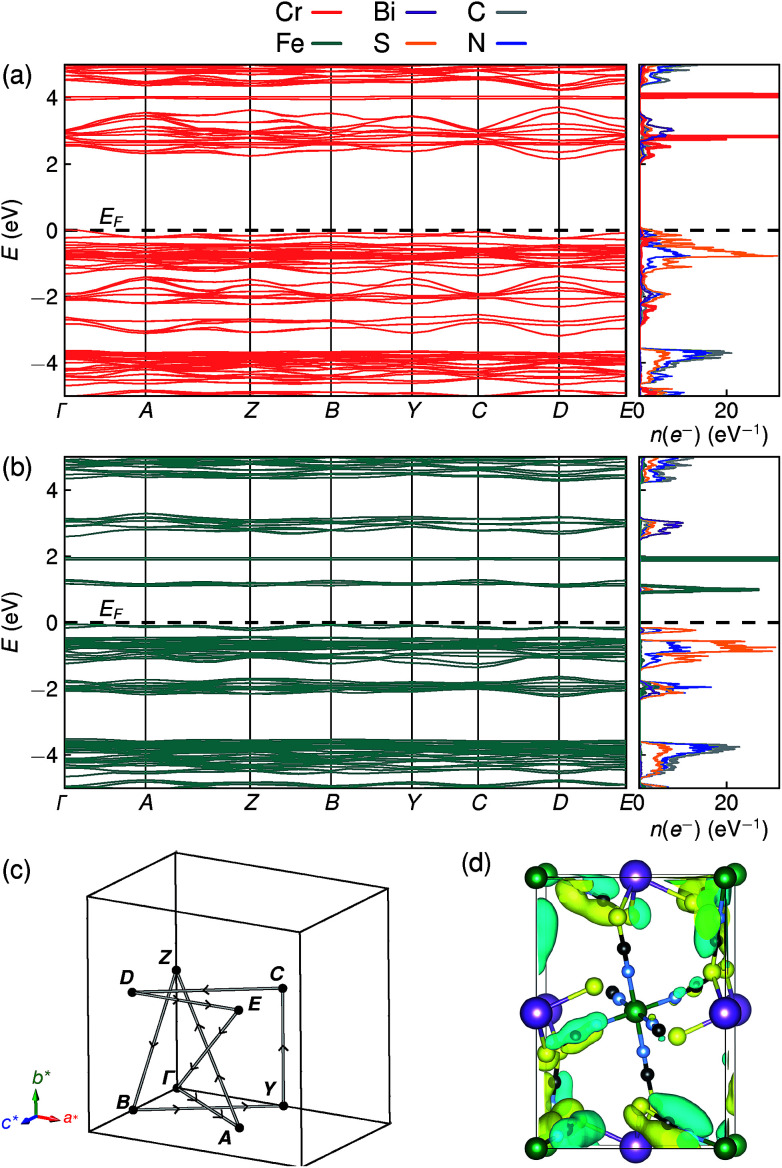
DFT calculated band structure and element-projected density of states for (a) Cr[Bi(SCN)_6_] and (b) Fe[Bi(SCN)_6_]. Both spin channels have been plotted at once, as the differences between them are minor. (c) Reciprocal space path used for band structure calculations. (d) Kohn–Sham orbital for Cr[Bi(SCN)_6_] at the valence band maximum, showing the localised nature of the valence band states, viewed along the [100] direction. Atoms are coloured as in Fig. 3.

Examination of the projected density of states suggests that the transition is primarily LMCT in character, as the states near the valence band maximum are primarily due to thiocyanate, and the conduction band minima states are mainly Fe for Fe[Bi(SCN)_6_] and primarily Cr and Bi for Cr[Bi(SCN)_6_] [Fig. 6a and b]. The narrow width of the calculated conduction and valence bands for both compounds indicates that the near band-edge states are highly localised, and so the effective masses of holes and electrons will be very large [Fig. 6a and b]. This is also borne out by examination of the Kohn–Sham orbitals at the valence band maximum, which are localised and confined to the ligands [Fig. 6(d)]. The lack of extensive delocalisation suggests that these frameworks would be better suited to applications where long-range electron transport is not essential, such as photocatalysis than as bulk semiconductors. Photoluminescence measurements on both single crystal and powder samples showed negligible fluorescence, suggesting that there is a high concentration of optical defects, borne out by the significant band-tails observed in both absorption and reflectance measurements. The slightly indirect nature of the band gap for these frameworks, particularly for Cr[Bi(SCN)_6_], could also be a contributory factor to the absence of observed fluorescence.

## 3 Conclusions

In this paper we report the synthesis, structure and optical properties of the first examples of a new family of molecule-based perovskite derived materials, transition metal hexathiocyanatobismuthmates M[Bi(SCN)_6_]. They are the first example of thiocyanate analogues of the cyanide Prussian blues, and to the best of our knowledge are the first molecular perovskite derived frameworks which incorporate heavy p-block cations. These compounds are vividly coloured, due to the presence of LMCT bands, and the higher optical absorption seen in these compounds suggests that thiocyanate-based frameworks may well be interesting materials to investigate for photocatalysis. We have also demonstrated the reversible uptake of water into Cr[Bi(SCN)_6_], which suggests it may be possible to incorporate other guests into this family of compounds. The chemical space opened up by these compounds, by substituting on the B site and introducing A site cations, and their unusual optical properties, suggests that thiocyanate perovskites could be an important family of molecular framework materials.

## Conflicts of interest

There are no conflicts of interest to declare.

## Supplementary Material

SC-010-C8SC04082F-s001

SC-010-C8SC04082F-s002

SC-010-C8SC04082F-s003

SC-010-C8SC04082F-s004
