# Supplementary material for: Strongly coloured thiocyanate frameworks with perovskite-analogue structures
Source: Chem Sci. 2018 Oct 26;10(3):793–801. doi: 10.1039/c8sc04082f (PMC6345348; doi:10.1039/c8sc04082f)
Supplement: SC-010-C8SC04082F-s001 [file SC-010-C8SC04082F-s001.pdf]

Electronic Supplementary Material (ESI) for Chemical Science.
This journal is © The Royal Society of Chemistry 2018

Strongly coloured thiocyanate frameworks with perovskite-analogue structures

Matthew J. Cliffe, Evan N. Keyzer, Matthew T. Dunstan, Shahab Ahmad, Michael F. L. De Volder, Felix Deschler Andrew J. Morris and Clare P. Grey*

October 24, 2018

Department of Chemistry, University of Cambridge,
Lensfield Road, Cambridge, CB2 1EW, U.K.
*To whom correspondence should be addressed; e-mail: cpg27@cam.ac.uk.

Contents

1 DFT+U details 3

2 Bi(NCS)$_3$ 10

3 Powder diffraction studies 12
3.1 Rietveld refinement of Cr[Bi(SCN)$_6$] 12
3.2 Rietveld refinement of Cr[Bi(SCN)$_6$] · H_2O 12
3.3 Sequential refinement of variable temperature data of Cr[Bi(SCN)$_6$] 12
3.4 Sequential refinement of variable temperature data of Cr[Bi(SCN)$_6$] · H_2O 12
3.5 Powder diffraction studies of Cr[Bi(SCN)$_6$] 13

4 UV-Vis studies 17

5 TGA 20

List of Figures

1 Variation in calculated optical (direct) band gap with the U parameter 3
2 Variation of FeBi(SCN)$_6$ DoS with U, spin polarisation indicated by positive or negative sign of density. 4
3 Variation of CrBi(SCN)$_6$ DoS with U, spin polarisation indicated by positive or negative sign of density. 5
4 Variation of CrBi(SCN)$_6$ DoS with U, spin polarisation indicated by positive or negative sign of density. 6
5 Variation of FeBi(SCN)$_6$ band structure with U. Spin polarisation indicated by colour of line. . . 7
6 Variation of CrBi(SCN)$_6$ band structure with U up to $U = 3.0$eV. Spin polarisation indicated by colour of line. 8
7 Variation of CrBi(SCN)$_6$ band structure with U greater than $U = 3.5$eV. Spin polarisation indicated by colour of line. 9

8 Crystal structure of $Bi(SCN)_3$, Bi in purple, N in blue, C in black, and S in yellow. Bi shown in polyhedral representation. 11
9 Hydration of anhydrous $Cr[Bi(SCN)_6]$. 13
10 Heatmap representation of variable temperature powder X-ray diffraction measurements of hydrous $Cr[Bi(SCN)_6] \cdot H_2O$. 14
11 Heatmap representation of variable temperature powder X-ray diffraction measurements of anhydrous $Cr[Bi(SCN)_6]$. 15
12 Side by side comparison of the heatmap representation of variable temperature powder X-ray diffraction measurements of anhydrous and hydrous $Cr[Bi(SCN)_6]$ highlighting the low Q region. 16
13 Comparison of powder diffuse reflectance (DR, purple, left) with integrating sphere absorption measurements carried out on a deposited film (IS, teal, right) for $Fe[Bi(SCN)_6]$. 17
14 Calculation of the optical band gap for $Fe[Bi(SCN)_6]$, using a Tauc plot assuming a direct band gap. 17
15 Calculation of the optical band gap for $Cr[Bi(SCN)_6]$, using a Tauc plot assuming a direct band gap. 18
16 Calculation of the thermal band gap for $Cr[Bi(SCN)_6]$, using a Tauc plot assuming a indirect band gap. 18
17 Calculation of the optical band gap for $Sc[Bi(SCN)_6]$, using a Tauc plot assuming a direct band gap. 19
18 Calculation of the thermal band gap for $Sc[Bi(SCN)_6]$, using a Tauc plot assuming a indirect band gap. 19
19 Full TGA mass loss curve for $Cr[Bi(SCN)_6]$ and $Cr[Bi(SCN)_6] \cdot H_2O$ measured under flowing N_2. 20
20 Top: Full TGA mass loss curve for $Fe[Bi(SCN)_6]$ measured under flowing N_2. Bottom: Temperature derivative of the TGA mass loss curve. The onset of the first mass loss (105 °) and the initial peak mass loss (130 °) are shown. 21

List of Tables

1 Bi-X bond distances 10

1 DFT+U details

In order to account for the known self-interaction error found in DFT calculations, we carried out calculations including a Hubbard U-parameter for a range of values, around those previously reported in the literature.[S1, S2] We found that although this did alter the energy of the metal d states, as expected, the band structure and density of states remained consistent over the full range explored. In the main text we present results for a value of U that produces a band gap consistent with that observed in the literature.

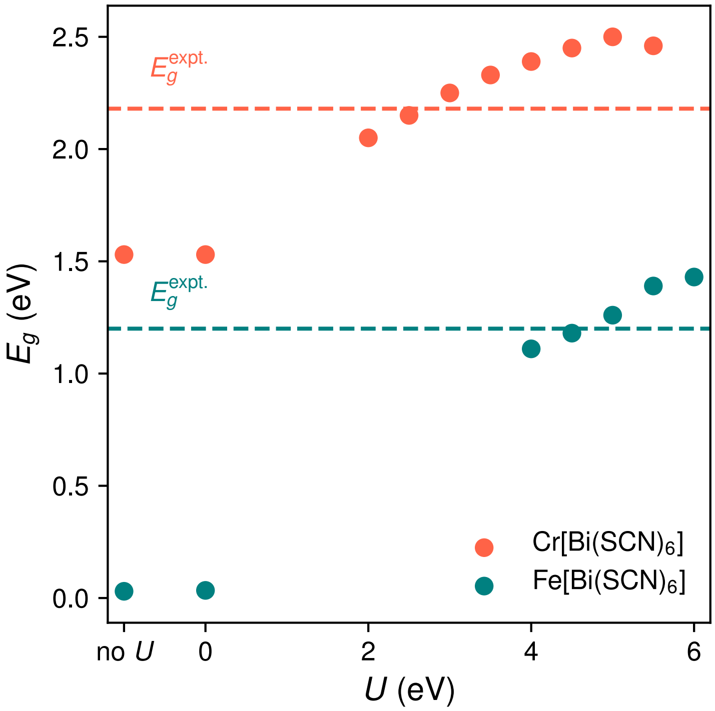

ESI Fig. 1: Variation in calculated optical (direct) band gap with the U parameter

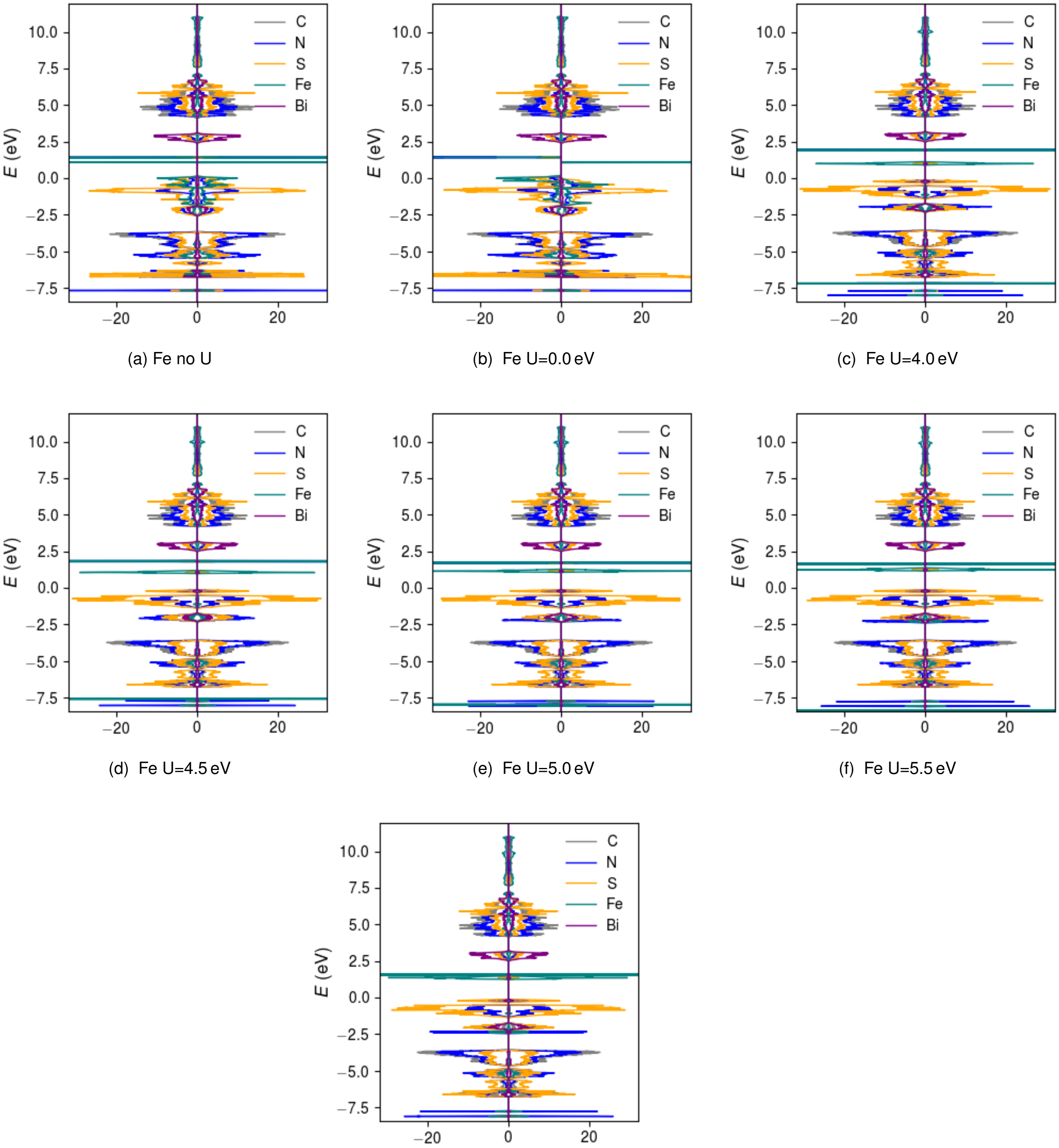

(a) Fe no U
(b) Fe U=0.0 eV
(c) Fe U=4.0 eV
(d) Fe U=4.5 eV
(e) Fe U=5.0 eV
(f) Fe U=5.5 eV
(g) Fe U=6.0 eV

ESI Fig. 2: Variation of $FeBi(SCN)_6$ DoS with U, spin polarisation indicated by positive or negative sign of density.

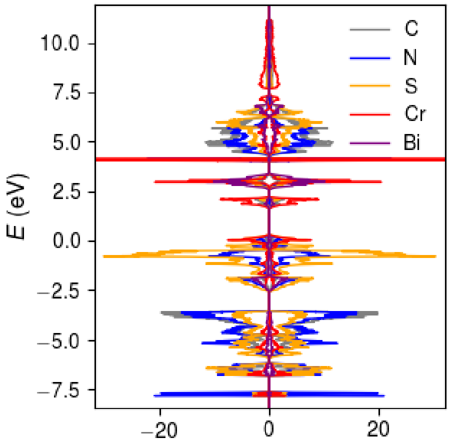

(a) no U

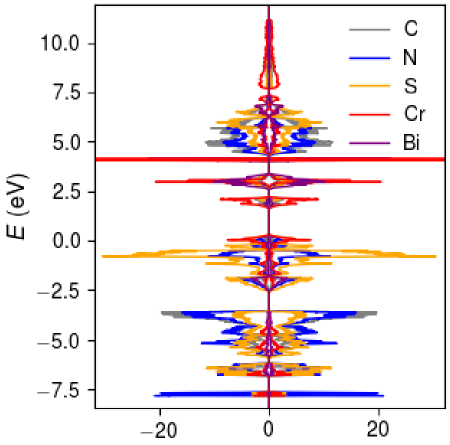

(b) Cr U=0.0 eV

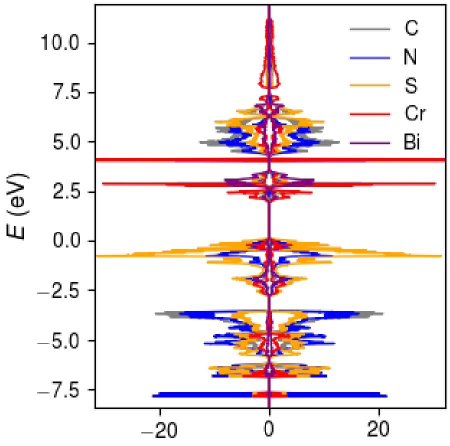

(c) Cr U=2.0 eV

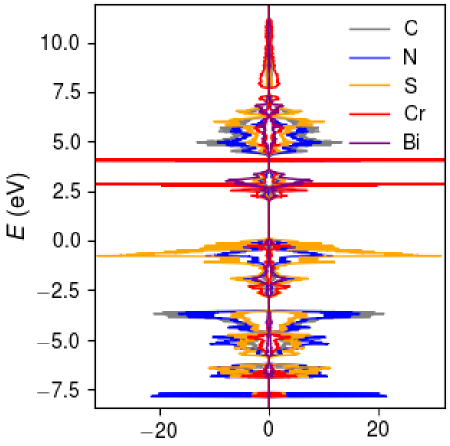

(d) Cr U=2.5 eV

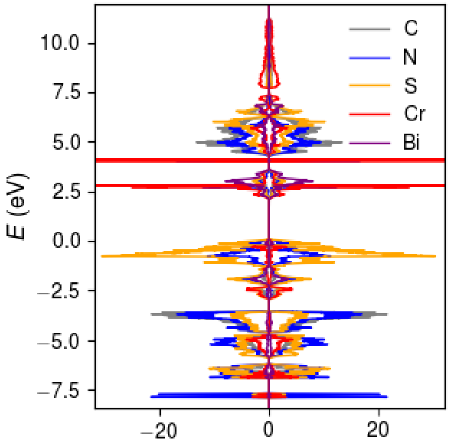

(e) Cr U=3.0 eV

ESI Fig. 3: Variation of $CrBi(SCN)_6$ DoS with U, spin polarisation indicated by positive or negative sign of density.

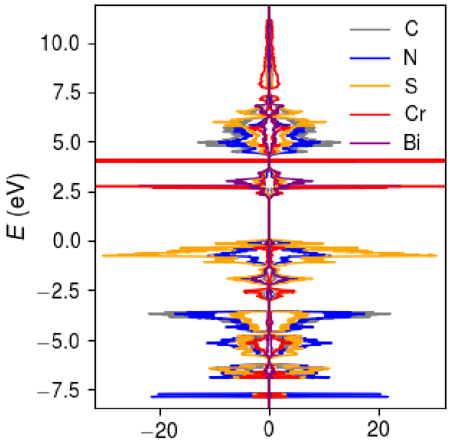

(a) Cr U=3.5 eV

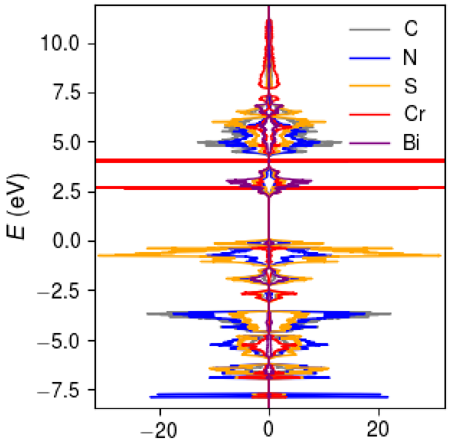

(b) Cr U=4.0 eV

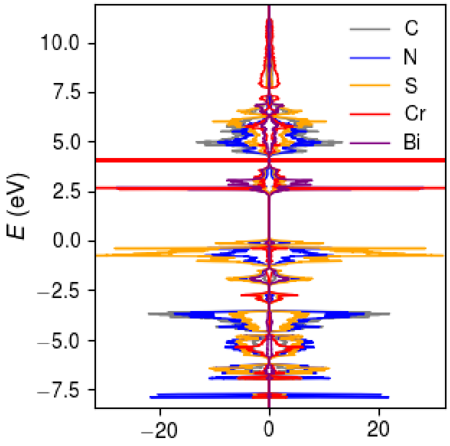

(c) Cr U=4.5 eV

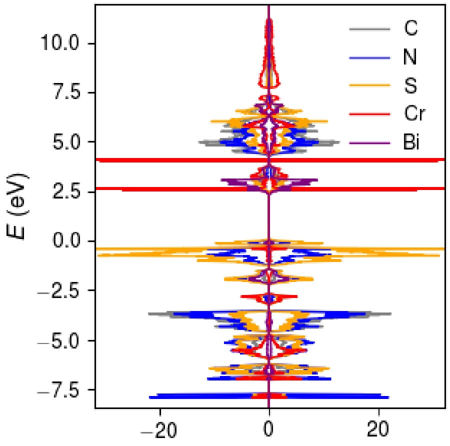

(d) Cr U=5.0 eV

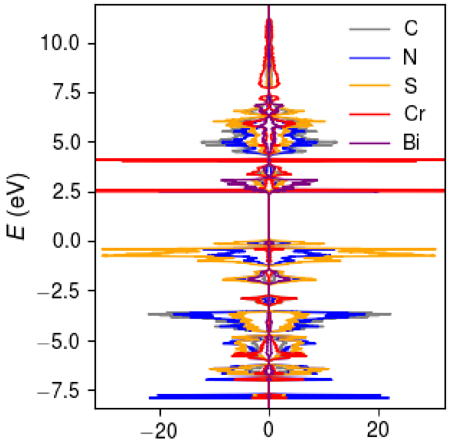

(e) Cr U=5.5 eV

ESI Fig. 4: Variation of $CrBi(SCN)_6$ DoS with U, spin polarisation indicated by positive or negative sign of density.

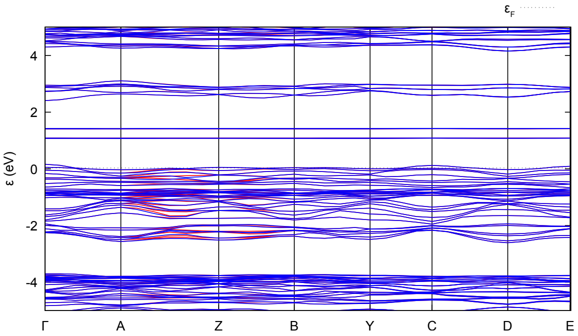

(a) no U

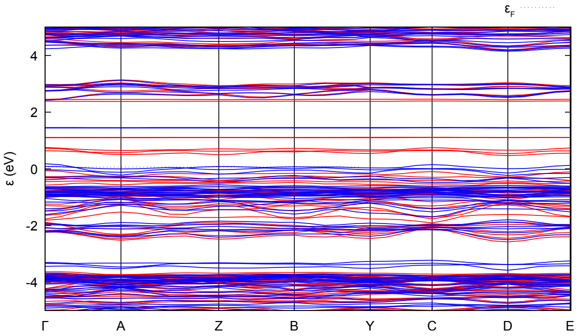

(b) U=0.0 eV

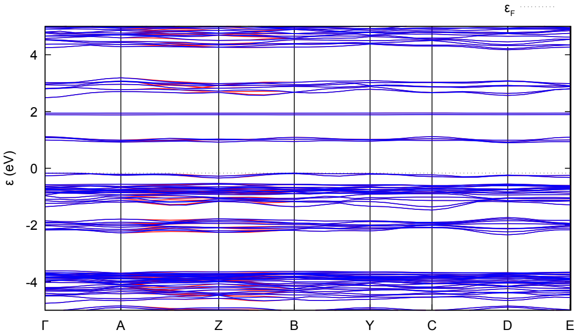

(c) U=4.0 eV

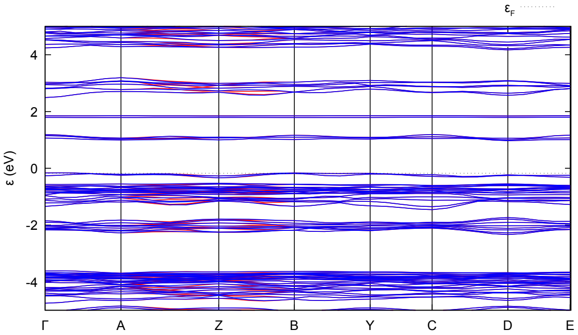

(d) U=4.5 eV

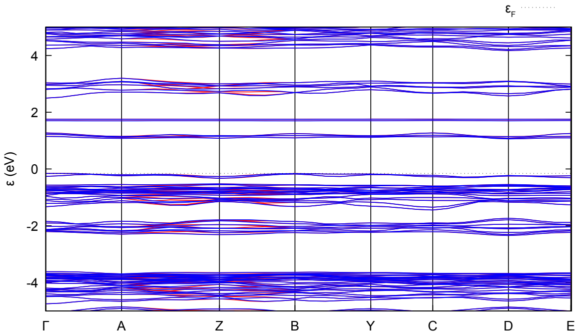

(e) U=5.0 eV

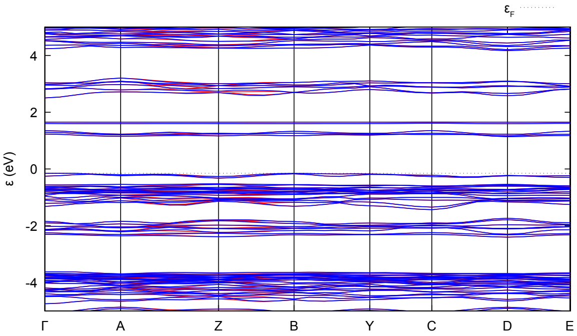

(f) U=5.5 eV

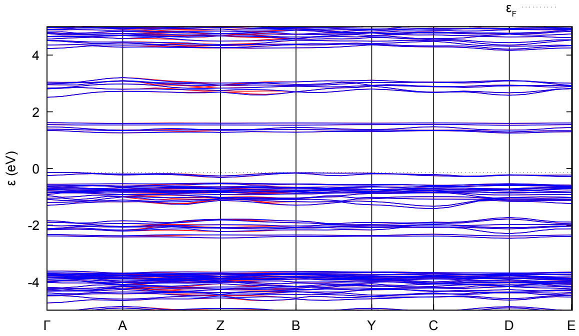

(g) U=6.0 eV

ESI Fig. 5: Variation of $FeBi(SCN)_6$ band structure with U. Spin polarisation indicated by colour of line.

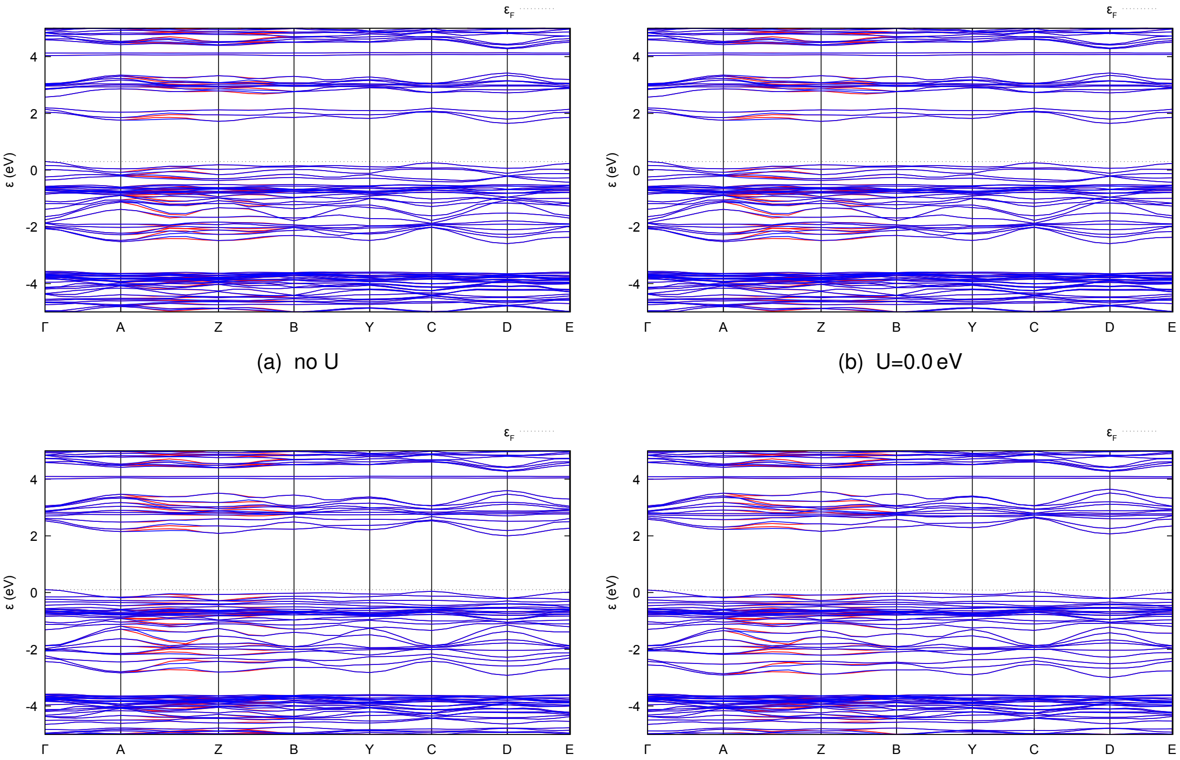

(a) no U

(b) U=0.0 eV

(c) U=2.5 eV

(d) U=3.0 eV

ESI Fig. 6: Variation of $CrBi(SCN)_6$ band structure with U up to $U = 3.0$eV. Spin polarisation indicated by colour of line.

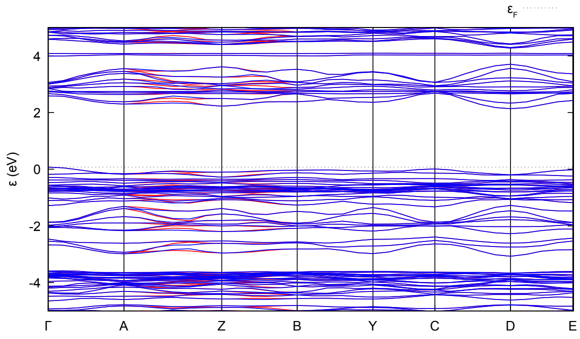

(a) U= 3.5 eV

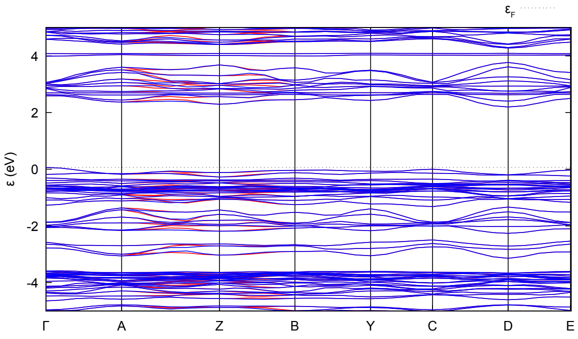

(b) U=4.0 eV

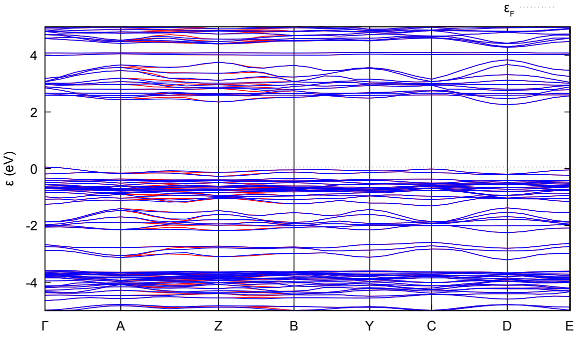

(c) U=4.5 eV

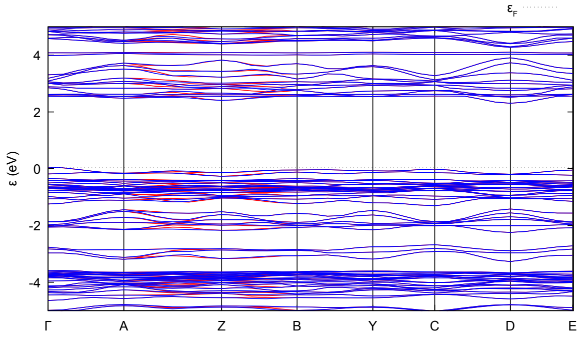

(d) U=5.0 eV

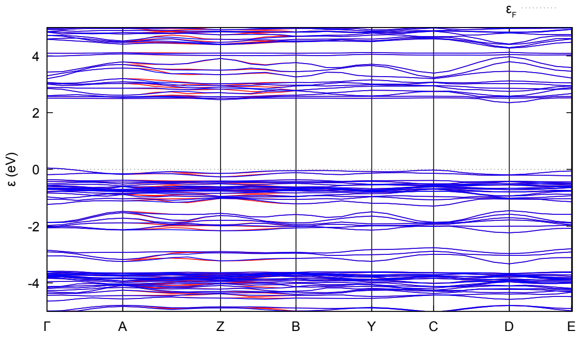

(e) U=5.5 eV

ESI Fig. 7: Variation of CrBi(SCN)$_6$ band structure with U greater than $U = 3.5$eV. Spin polarisation indicated by colour of line.

2 $Bi(NCS)_3$

During the synthesis of $Fe[Bi(SCN)_6]$ we were able to obtain single crystals of $Bi(SCN)_3$ from the evaporation of $H_3[Bi(SCN)_6]$ solutions in water. Here we briefly describe the structure of this new material. $Bi(SCN)_3$ crystallises in a low symmetry triclinic $P\bar{1}$ structure containing two distinct Bi^{3+} and six distinct NCS^- sites. The bonding around each Bi^{3+} is very irregular, Bi01 forming short bonds to two sulfur atoms (2.68 Å) and two nitrogen atoms (2.4 Å) and Bi02 forming short bonds to two sulfurs and one nitrogen. If only these short bonds are considered Bi01 is roughly square pyramidal forming a molecular dimer $(NCS)_2Bi(NCS)_2Bi(NCS)_2$ and Bi02 is a triangular pyramid forming an isolated $Bi(SCN)_3$ molecular. When longer interactions are included, these molecular species connect into a three dimensional framework structure formed from dimers of distorted $Bi01N_4S_4$ polyhedron face sharing with a distorted octahedral $Bi02N_3S_3$ which are linked through NCS to other dimers. This three dimensional network is illustrated in ESI Figs. 8.

ESI Table 1: Bi-X bond distances

Bi	X	r(Å)	Bi	X	r(Å)
Bi01	S003	2.679(3)	Bi02	S004	3.005(3)
Bi01	S005	2.686(3)	Bi02	S006	2.699(3)
Bi01	N00C	2.434(9)	Bi02	S007	3.059(3)
Bi01	N00F	2.462(9)	Bi02	S008	2.677(3)
Bi01	N00G	2.674(11)	Bi02	N00A	2.345(9)
			Bi02	N00D	2.506(9)

Estimated standard error in parantheses.

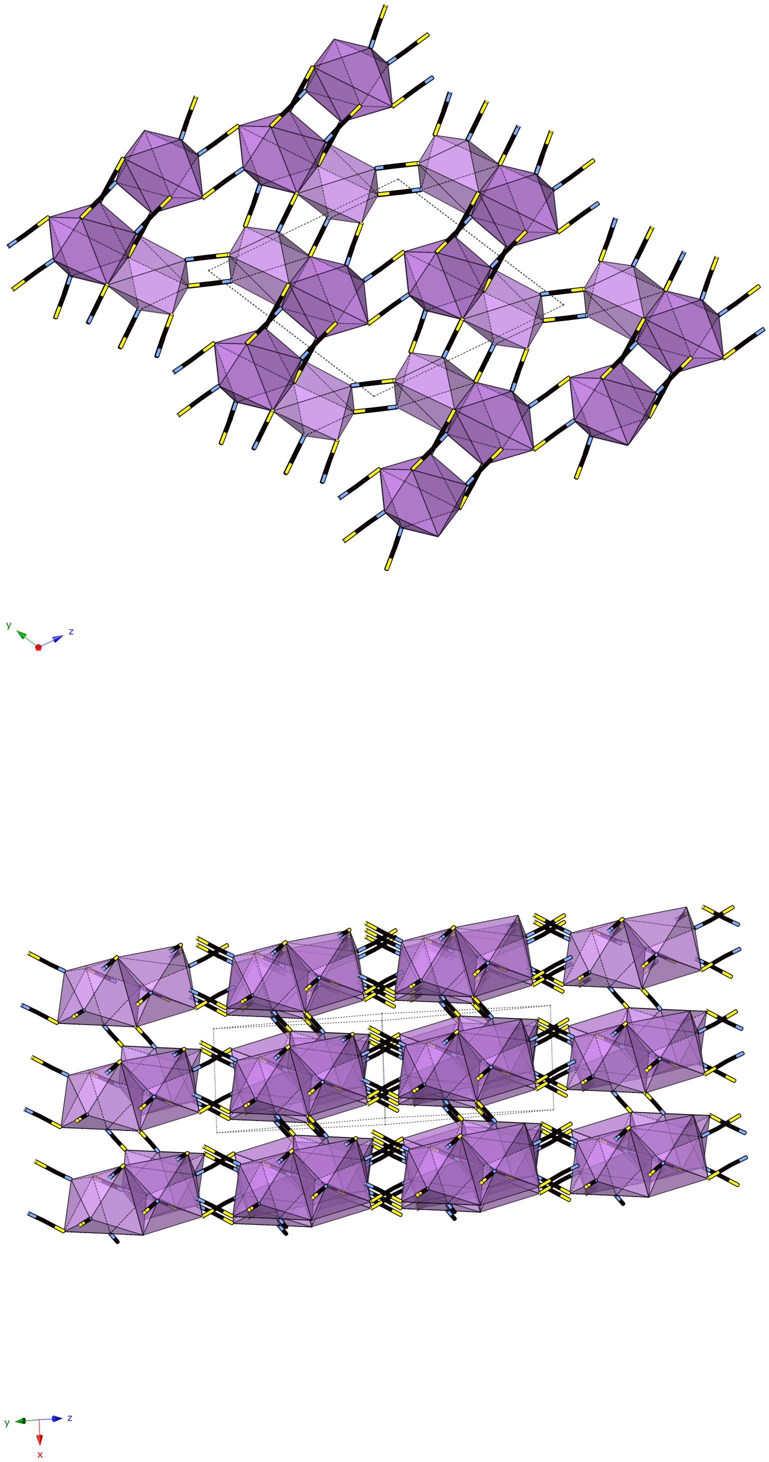

ESI Fig. 8: Crystal structure of $Bi(SCN)_3$, Bi in purple, N in blue, C in black, and S in yellow. Bi shown in polyhedral representation.

3 Powder diffraction studies

3.1 Rietveld refinement of $Cr[Bi(SCN)_6]$

Rietveld refinement of $Cr[Bi(SCN)_6]$ was carried out from the single crystal structure of $Fe[Bi(SCN)_6]$, with Cr substituted for Fe. The $Cr(NCS)_6$ octahedron was constrained as a rigid body, with the bond angles retained but the lengths fixed to $d_{\mathrm{CN}} = 1.15$ Å, $d_{\mathrm{NS}} = 1.65$ Å, $d_{\mathrm{CrN}} = 2.0$ Å and uniform isotropic atomic displacement parameters for the entire octahedron. The background terms were refined using a Chebyschev polynomial, and lattice parameters, rotation of the rigid octahedron, and isotropic displacement parameters were allowed to refine freely, along with crystallographic size and strain (anisotropically modelled using fourth-order spherical harmonics). Refinement statistics: $R_{wp} = 10.998$, $R_{\mathrm{Bragg}} = 3.50$, goodness of fit = 1.871.

3.2 Rietveld refinement of $Cr[Bi(SCN)_6] \cdot H_2O$

Rietveld refinement of $Cr[Bi(SCN)_6] \cdot H_2O$ was carried out from the single crystal structure of $Fe[Bi(SCN)_6]$, with Cr substituted for Fe. The $Cr(NCS)_6$ octahedron was constrained as a rigid body, with the bond angles retained but the lengths fixed to $d_{\mathrm{CN}} = 1.15$ Å, $d_{\mathrm{NS}} = 1.65$ Å, $d_{\mathrm{CrN}} = 2.0$ Å and uniform isotropic atomic displacement parameters for the entire octahedron. The background terms were refined using a Chebyschev polynomial, and lattice parameters, rotation of the rigid octahedron, and isotropic displacement parameters were allowed to refine freely, along with crystallographic size and strain (anisotropically modelled using fourth-order spherical harmonics). The guest oxygen was added at a position located from the void map produced in Mercury, its occupancy fixed the occupancy determined from TGA (i.e. fully occupied), and then its position and isotropic atomic displacement parameter allowed to refine freely. The hydrogen atoms were not included as they could not be located. Without the guest water included, the refinement statistics are appreciable worse: $R_{wp} = 3.706$ instead of $R_{wp} = 3.162$. Refinement statistics: $R_{wp} = 3.162$, $R_{\mathrm{Bragg}} = 4.216$, goodness of fit = 7.542.

3.3 Sequential refinement of variable temperature data of $Cr[Bi(SCN)_6]$

Lattice parameters were extracted using Rietveld refinement of laboratory powder X-ray diffraction data. Due to the low resolution of the data sets and the acute pseudo symmetry, Pawley refinement proved unable to reliably distinguish the three crystallographic axes. We therefore resorted to Rietveld refinement, where the atomic positions were kept fixed of $Fe[Bi(SCN)_6]$, to better deconvolute overlapping peaks. The background, strain, and lattice parameters were allowed to refine freely throughout the refinement.

3.4 Sequential refinement of variable temperature data of $Cr[Bi(SCN)_6] \cdot H_2O$

Lattice parameters were extracted using Pawley refinement of high resolution synchrotron powder X-ray diffraction data. Above 150 °C, the dehydration of the structure set in and the resulting combination of pseudosymmetry and phase mixtures prevented us from carrying out reliable quantitative fits of the data sets. The background, strain, and lattice parameters were allowed to refine freely throughout the refinement.

3.5 Powder diffraction studies of $Cr[Bi(SCN)_6]$

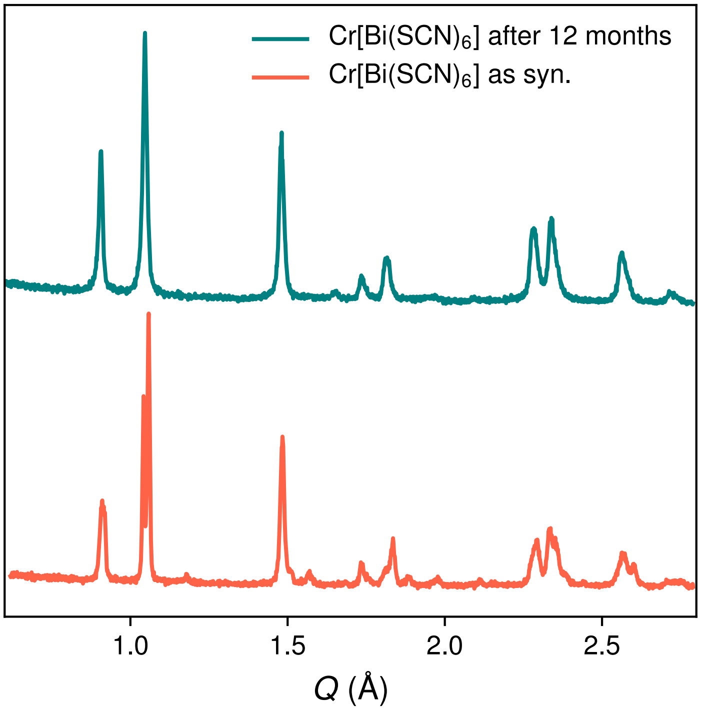

ESI Fig. 9: Hydration of anhydrous $Cr[Bi(SCN)_6]$.

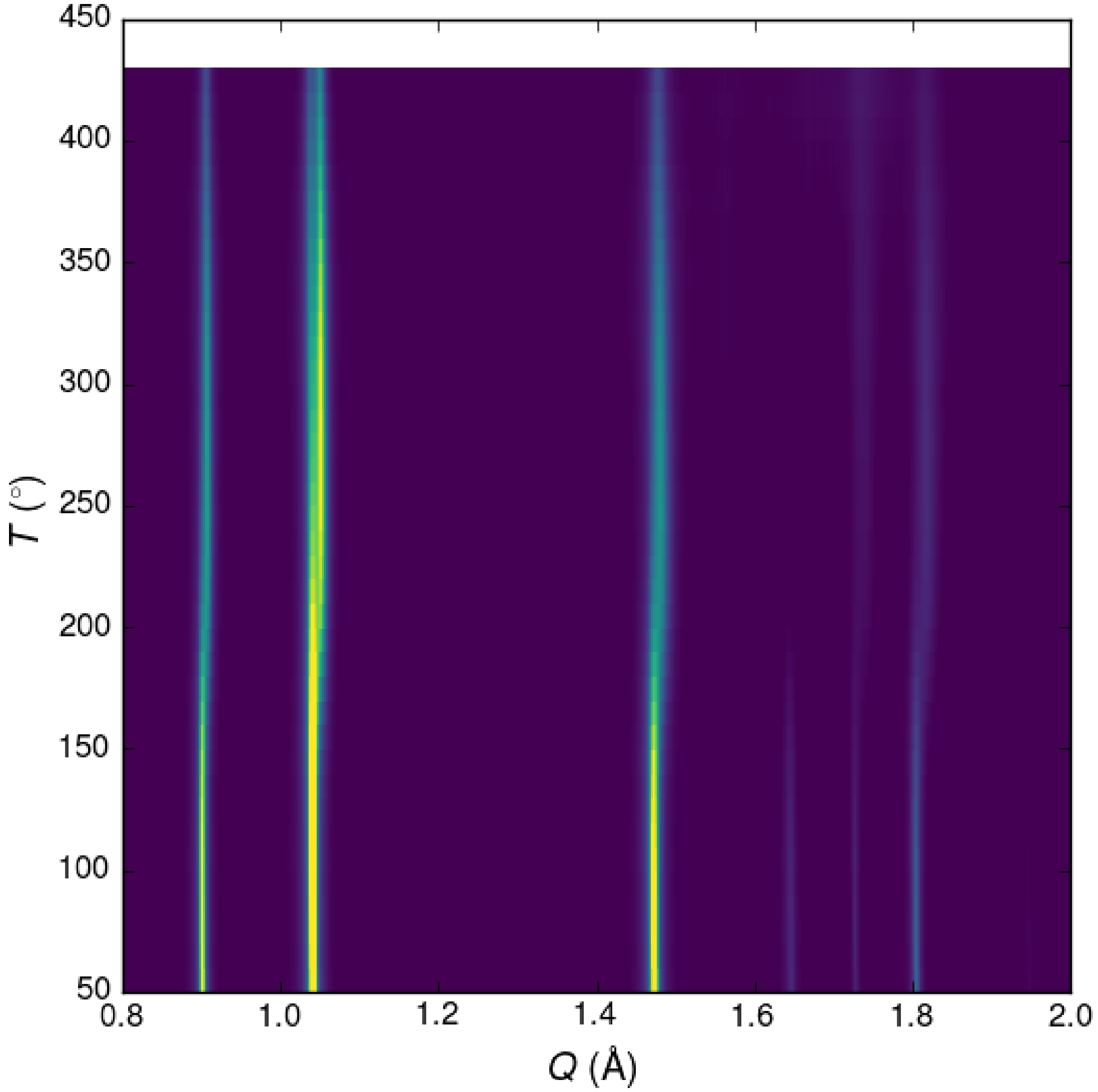

ESI Fig. 10: Heatmap representation of variable temperature powder X-ray diffraction measurements of hydrous $Cr[Bi(SCN)_6] \cdot H_2O$.

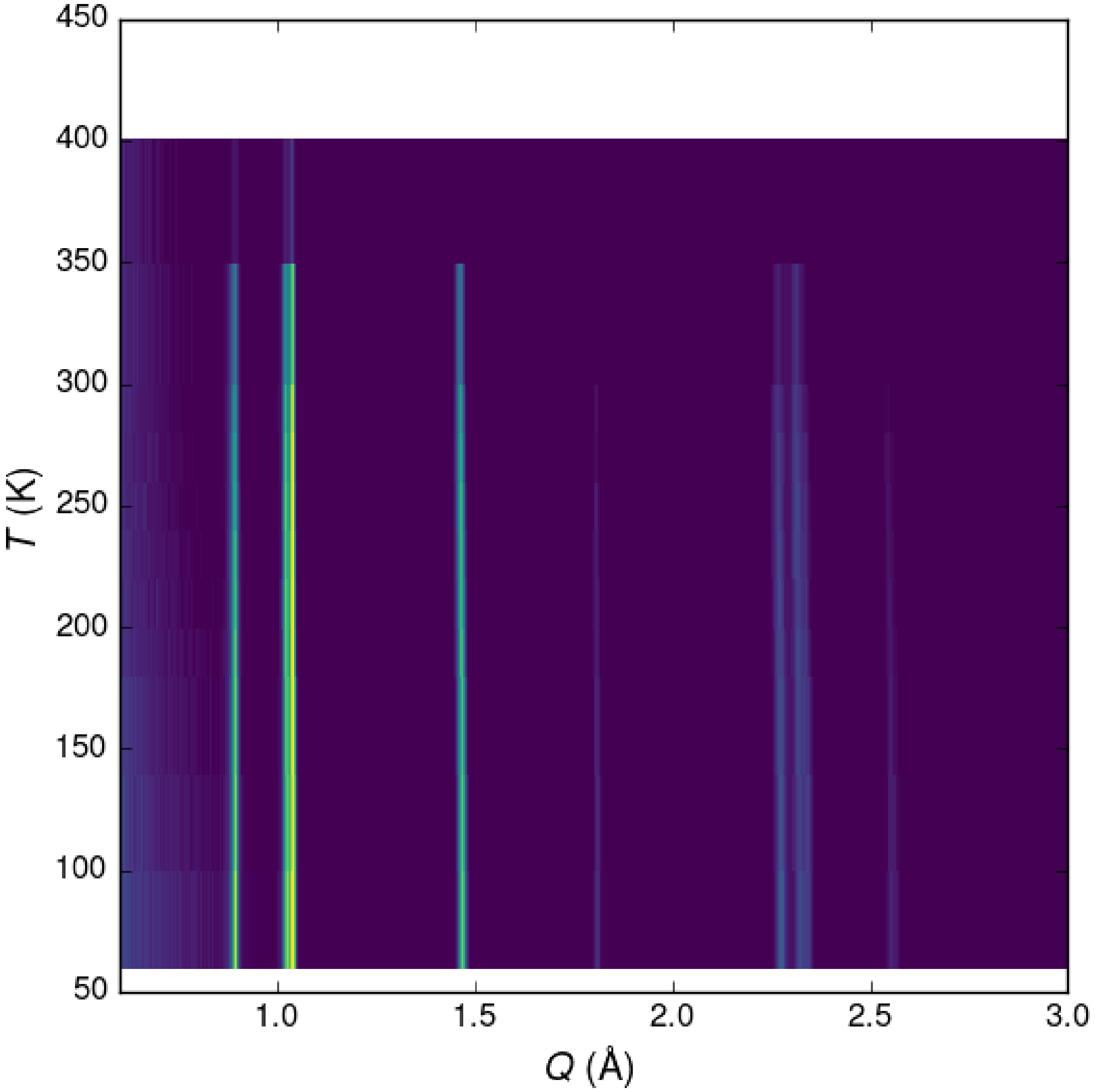

ESI Fig. 11: Heatmap representation of variable temperature powder X-ray diffraction measurements of anhydrous $Cr[Bi(SCN)_6]$.

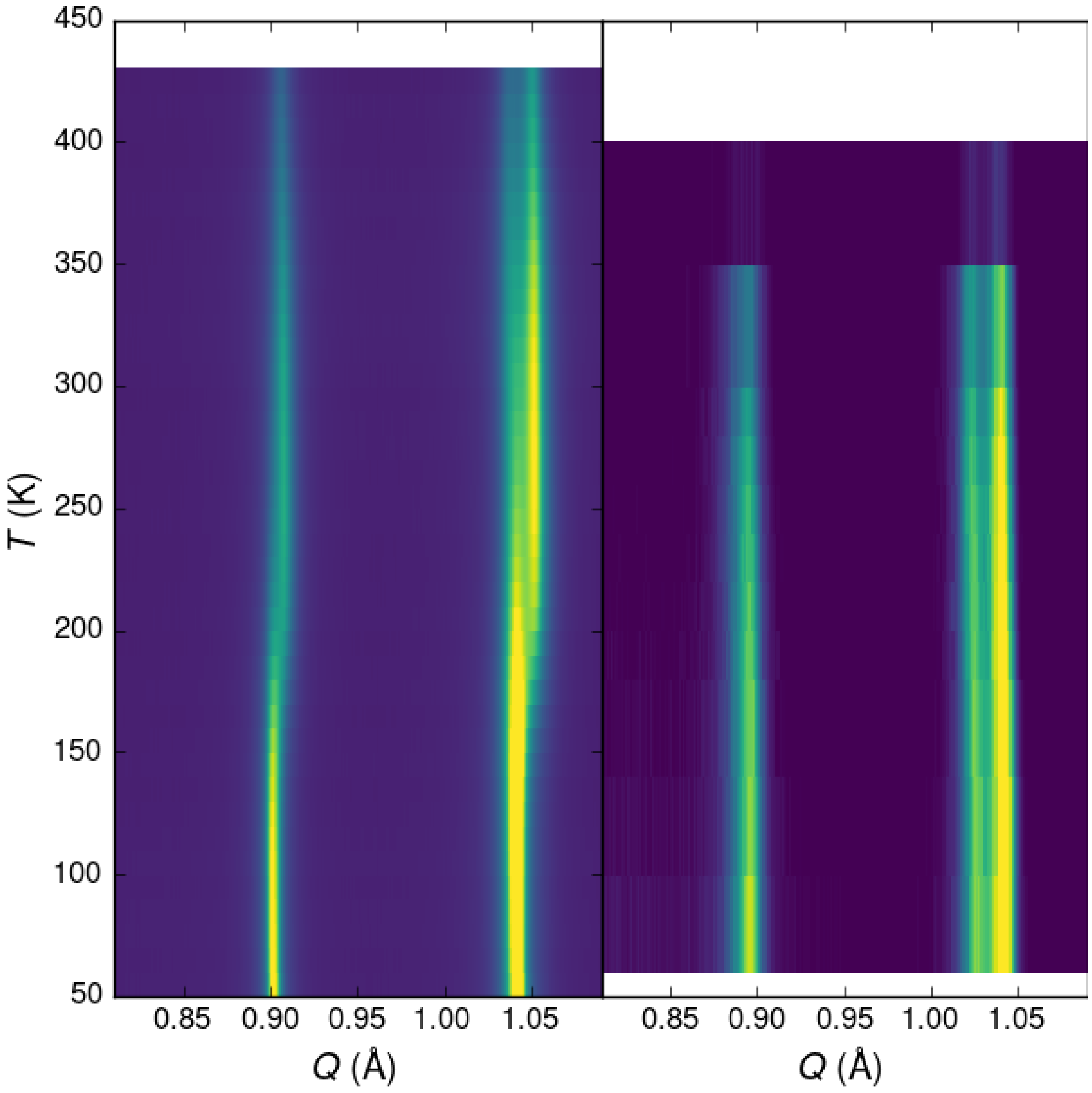

ESI Fig. 12: Side by side comparison of the heatmap representation of variable temperature powder X-ray diffraction measurements of anhydrous and hydrous $Cr[Bi(SCN)_6]$ highlighting the low Q region.

4 UV-Vis studies

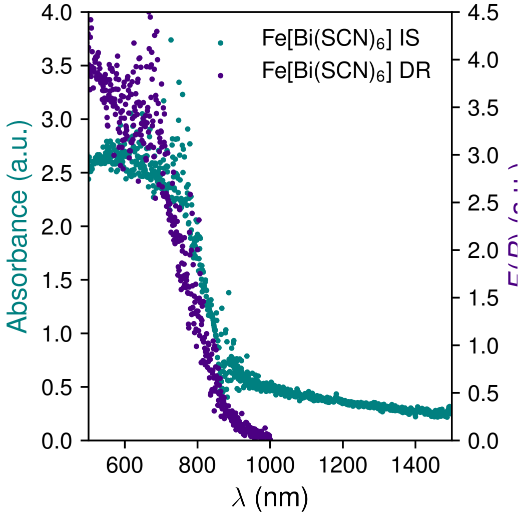

ESI Fig. 13: Comparison of powder diffuse reflectance (DR, purple, left) with integrating sphere absorption measurements carried out on a deposited film (IS, teal, right) for $Fe[Bi(SCN)_6]$.

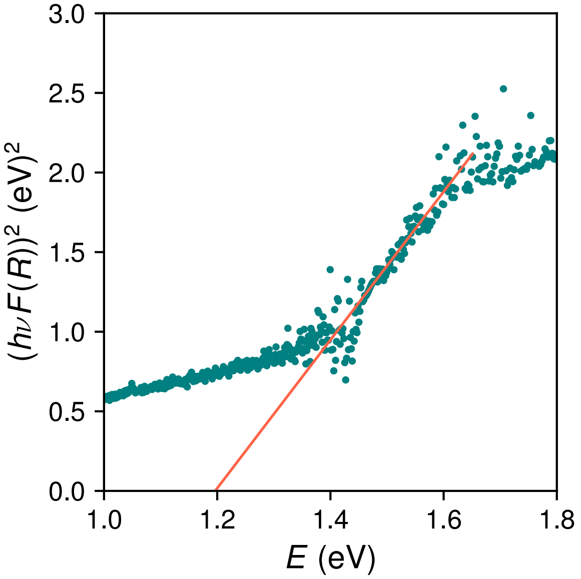

ESI Fig. 14: Calculation of the optical band gap for $Fe[Bi(SCN)_6]$, using a Tauc plot assuming a direct band gap.

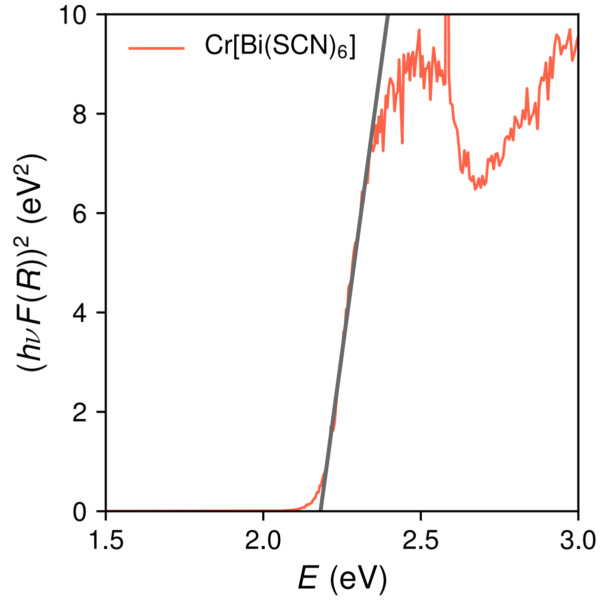

ESI Fig. 15: Calculation of the optical band gap for Cr[Bi(SCN)$_6$], using a Tauc plot assuming a direct band gap.

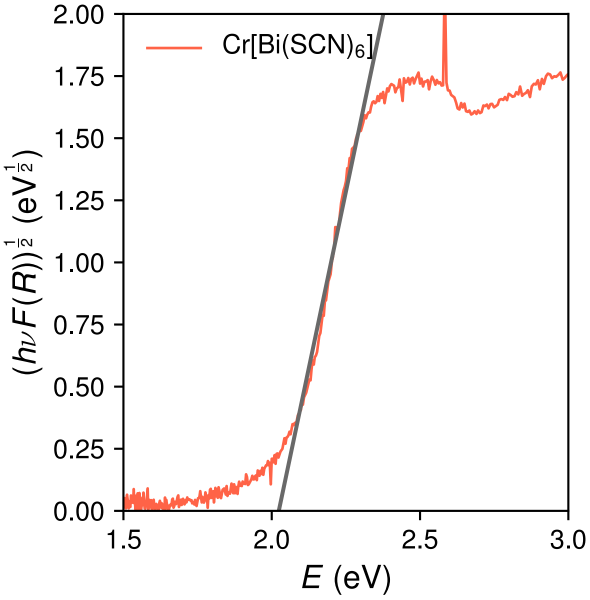

ESI Fig. 16: Calculation of the thermal band gap for Cr[Bi(SCN)$_6$], using a Tauc plot assuming a indirect band gap.

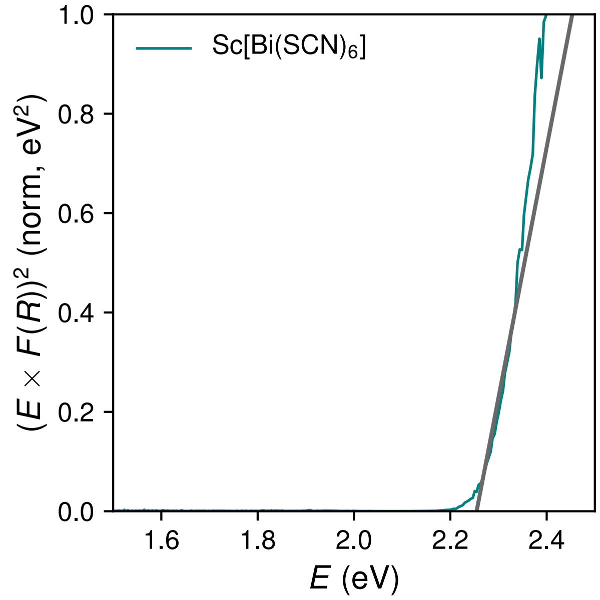

ESI Fig. 17: Calculation of the optical band gap for $Sc[Bi(SCN)_6]$, using a Tauc plot assuming a direct band gap.

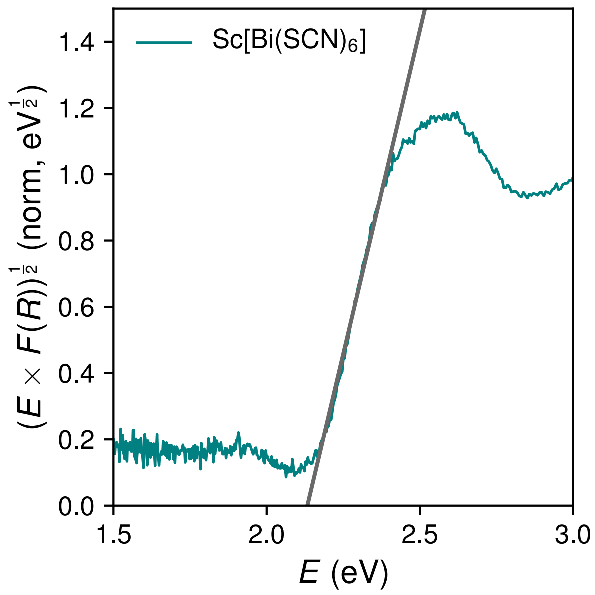

ESI Fig. 18: Calculation of the thermal band gap for $Sc[Bi(SCN)_6]$, using a Tauc plot assuming a indirect band gap.

5 TGA

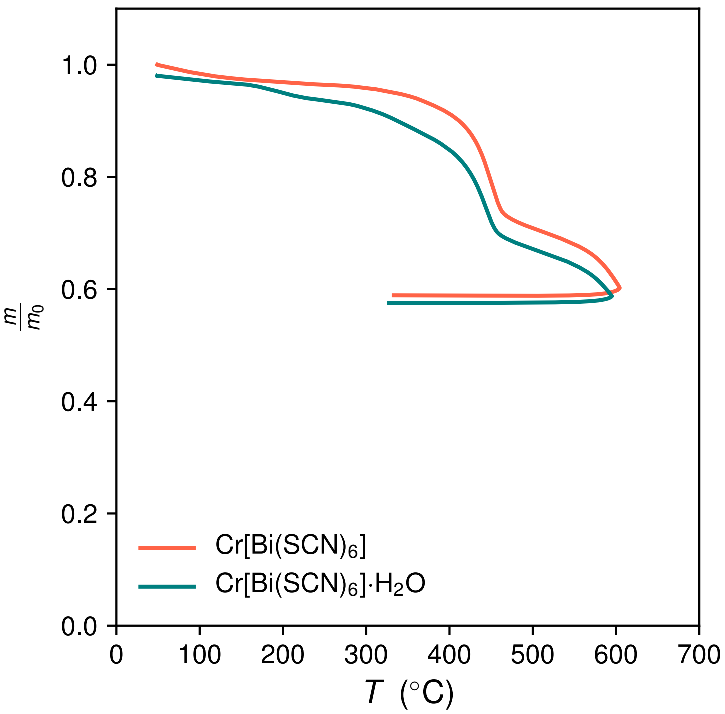

ESI Fig. 19: Full TGA mass loss curve for $Cr[Bi(SCN)_6]$ and $Cr[Bi(SCN)_6] \cdot H_2O$ measured under flowing N_2.

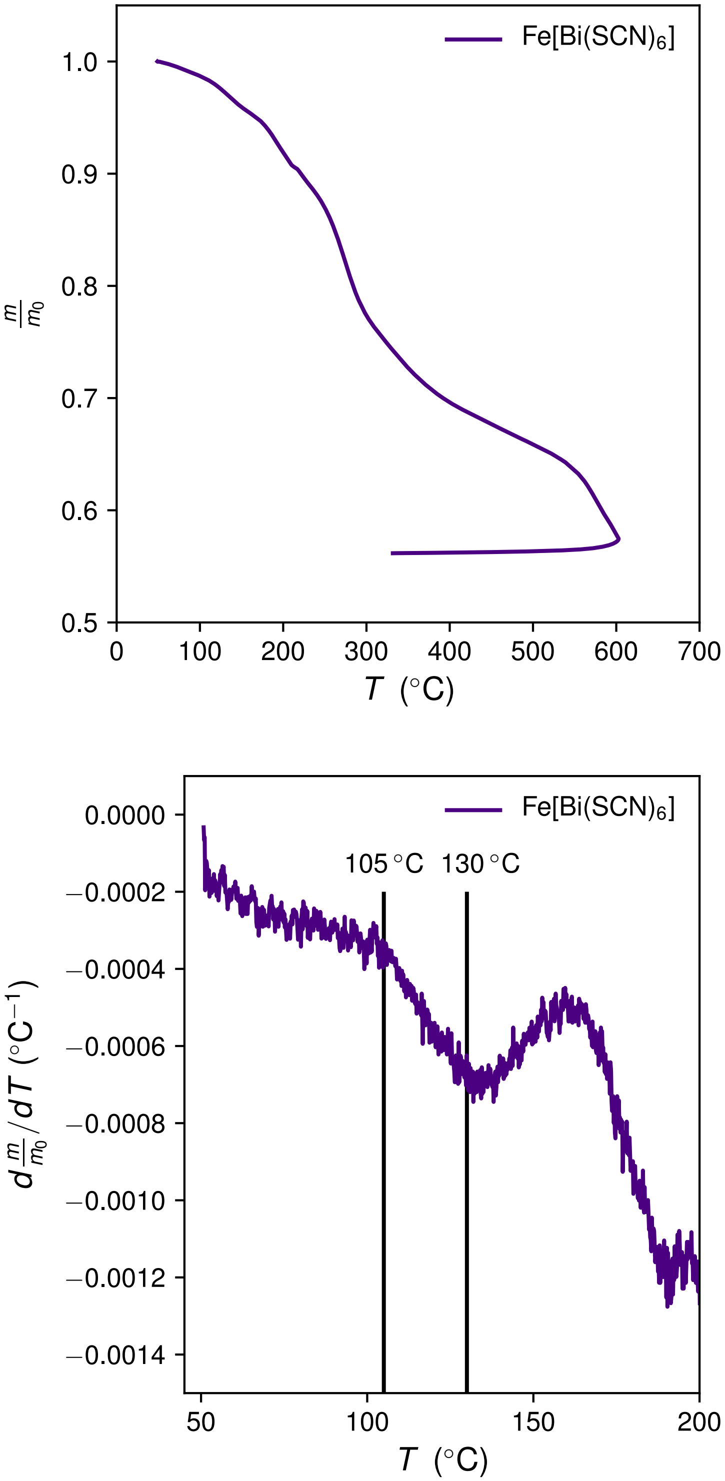

ESI Fig. 20: Top: Full TGA mass loss curve for $Fe[Bi(SCN)_6]$ measured under flowing N_2. Bottom: Temperature derivative of the TGA mass loss curve. The onset of the first mass loss (105 °) and the initial peak mass loss (130 °) are shown.

References

(S1) D. O. Scanlon, *et al.*, *Phys. Rev. B* **79**, 035101 (2009).
(S2) M. Cococcioni, S. de Gironcoli, *Physical Review B* **71** (2005).
